# Gas Phase Computational Study of Diclofenac Adsorption on Chitosan Materials

**DOI:** 10.3390/molecules25112549

**Published:** 2020-05-30

**Authors:** Anna Kaczmarek-Kędziera

**Affiliations:** Faculty of Chemistry, Nicolaus Copernicus University in Toruń, Gagarina 7, 87-100 Toruń, Poland; teoadk@chem.umk.pl

**Keywords:** chitosan, diclofenac, non-steroidal anti-inflammatory drugs, DFT calculations, interaction energy, SAPT

## Abstract

Environmental pollution with non-steroidal anti-inflammatory drugs and their metabolites exposes living organisms on their long-lasting, damaging influence. Hence, the ways of non-steroidal anti-inflammatory drugs (NSAIDs) removal from soils and wastewater is sought for. Among the potential adsorbents, biopolymers are employed for their good availability, biodegradability and low costs. The first available theoretical modeling study of the interactions of diclofenac with models of pristine chitosan and its modified chains is presented here. Supermolecular interaction energy in chitosan:drug complexes is compared with the the mutual attraction of the chitosan dimers. Supermolecular interaction energy for the chitosan-diclofenac complexes is significantly lower than the mutual interaction between two chitosan chains, suggesting that the diclofenac molecule will encounter problems when penetrating into the chitosan material. However, its surface adsorption is feasible due to a large number of hydrogen bond donors and acceptors both in biopolymer and in diclofenac. Modification of chitosan material introducing long-distanced amino groups significantly influences the intramolecular interactions within a single polymer chain, thus blocking the access of diclofenac to the biopolymer backbone. The strongest attraction between two chitosan chains with two long-distanced amino groups can exceed 120 kcal/mol, while the modified chitosan:diclofenac interaction remains of the order of 20 to 40 kcal/mol.

## 1. Introduction

Non-steroidal anti-inflammatory drugs (NSAIDs) are the most popular over-the-counter pharmaceuticals available in numerous formulations (injections, tablets, ointments). Apart from the applications in frequent ailments such as headache, fever, muscle pains or dysmenorrhea, they are administered in chronic diseases (for example rheumatoid arthritis). This causes the uncontrollable contamination of soils and wastewater both by NSAIDs and their metabolites. Although these pollutions exist in environment only in ppb or ppt concentrations, the permanent exposition for their action can have enormous impact on living organisms. Therefore, the new effective ways of detection and removal of these pollutions are sought for. Among various methods such as hydrolysis, biodegradation or chemical oxidation, adsorption on different materials appears particularly interesting for this purpose, since it allows to remove not only NSAIDs themselves but also their harmful metabolites. As the adsorbents, silicates, zeolites or carbon materials are widely used, however the easy access and low prices direct the focus also to biopolymers such as chitosan (CS).

Chitosan structure itself has been a subject of the extensive investigation in the previous years as the carbohydrates gain more and more interest in various branches of science and technology. The precise knowledge of the 3D structure of this polysaccharide would allow to predict mechanisms of its action and actual interaction in living organisms. However, due to the large chain flexibility and varying lengths (molecular weight) together with the susceptibility to the influence of the pH changes and solution ionic strengths, the definite arrangement of the chitosan chain is extremely difficult to capture with the available techniques (mostly X-ray and NMR) and anyway may be non-persistent under the particular desired conditions [1,2,3]. Chitosan is known to adopt one of five different helical arrangements and the stability of the intrachain O3H${}_{\left(n\right)}$...O5${}_{\left(n+1\right)}$ hydrogen bonds significantly favors the 2-fold helix [2].

The wide range of interest in chitosan for pharmaceutical, biomedical and biotechnological purposes arise from its nontoxicity, biocompatibility, biodegradability and large abundance in nature. Chitosan is readily applied in the drug delivery systems for its biocompatibility and antiulcer and antiacid characteristics. Due to the numerous amino and hydroxyl groups, it can easily bind other chemicals such as drugs via establishing weak intermolecular interactions that significantly stabilize molecular complexes. Numerous experimental studies confirm the applicability of chitosan for controlled release of diclofenac [4,5,6,7,8]. The tissue engineering on the other hand exploits the porous structure and gel-forming properties of chitosan together with its high affinity to in vivo macromolecules [9].

The above mentioned properties of chitosan together with its adsorption potential first described for metal ions in a series of papers by Muzzarelli [10,11,12,13] offers also an attractive alternative for application of chitosan in environmental pollution removal, dye industry, development of chromatographic supports or drug transport processes [14,15,16]. These can lead to the wide utilization of chitosan as effective adsorbents in the wastewater purification. However, due to the relatively low surface-to-volume ratio, high crystallinity and low mechanical strength, pristine chitosan occurs to be significantly inferior with respect to its modified derivatives.

According to the previous experimental survey, the chemical modification of the biopolymers either by cross-linking or grafting can lead to the production of highly functionalized materials of potential applications in medicine and biotechnology, improved solubility, increased surface-to-volume ratio and in consequence high absorption capacity [17,18,19,20,21,22,23,24,25,26]. Additionally, also the chitosan can be deposited on the inorganic core surface of desired characteristics such as Fe${}_{3}$O${}_{4}$ exhibiting strong magnetic properties [14,27]. The added value of this material is that the polymer shell protects the core from oxidization and magnetism loss and therefore after the interaction between the pollution and chitosan functional groups occurs, they can be simply removed from environment with magnet. Such magnetic chitosan materials were successfully applied for carbamazepine or diclofenac sorption from wastewater [14,17,28].

The grafting can be carried out on the way of a functionalization of the side hydroxyl and/or amino groups in the main polymer chain. Of particular interest are the newly introduced amino groups placed on the terminus of the long alkyl substituents—this makes them more exposed for the interaction with any reagents from the reaction environment and prone for biomedical applications such as enzyme immobilization or interaction with chemical species containing carboxyl group [14,18,27,29]. The initial analysis of the properties of the chitosan derivatives obtained in our group either by the reactions with glutaraldehyde aqueous solution of ethylenediamine, CS(NH${}_{2}$) (amino groups functionalization) or by epichlorohydrin treatment in alkali solution, next—glutaraldehyde and finally ethylenediamine reaction, CS(NH${}_{2}$)${}_{2}$ and CS(NH${}_{2}$)${}_{3}$, show non-monotonic behavior of these three derivatives with respect to various properties such as surface area of the obtained material, contact angle or the ability of human serum albumin immobilization [27]. In order to get insight into this non apparent nature and properties of the analyzed chitosan derivatives, the molecular dynamic (MD) simulation of the chains with 20 units was performed that shows a different H-bond pattern in these systems. In order to reproduce the natural environment in the living organisms well, the polymer was solvated in TIP3P water, surrounding the chain with a layer of minimum 30 Å. Due to the long chain, such a simulation included up to 150,000 atoms. Together with the prior careful minimization and equilibration procedure, the 100 ns of a production run for such a large system allows to obtain the results valuable from the statistical point of view and reproducing well the polymer chain behavior solvated in water. As could be expected, the nature of the CS(NH${}_{2}$) chain is determined by the O–H...O hydrogen bonds involving the hydroxyl groups in the main chain that remain unaltered during the chemical modification process. CS(NH${}_{2}$) is also most flexible among all the analyzed polymers. Similar situation is observed in CS(NH${}_{2}$)${}_{3}$. However, since the simulation was performed with the consideration of the deacylation degree of 80% in order to correspond well to the experimental materials, in the case of this chain most of the O–H...O hydrogen bonds involve the chitin units [27]. On the other hand, in CS(NH${}_{2}$)${}_{2}$, where the hydroxyl groups are substituted by the lengthy side chains, the only possible hydrogen bonds are of the N–H...O type. These are not observed in CS(NH${}_{2}$) and CS(NH${}_{2}$)${}_{3}$ due to the absence of the main chain amino groups and impose the decisive influence on the material properties, forming dense aggregates and therefore reducing the possibility of its biomedical application.

The broad and nonuniform set of NSAIDs cover the chemicals differing in structure and activity, while their general action is inhibition of cyclooxygenase COX-1 and/or COX-2 enzyme. Among the most popular members of NSAIDs family, diclofenac (2-(2,6-dichloranilino) phenylacetic acid, denoted later on as DFH) should be mentioned as designed in 1970s COX-2 inhibitor revealing also the potential anti-cancer action. It fulfills the structural and property assumptions for best anti-rheumatic COX-2 inhibitors: acidity constant $pK_{a}=4.0$, partition coefficient $\log_{p}\left(octanol/water\right)=13.4$ and two twisted aromatic rings [30]. Due to the weak solubility of the diclofenac in acidic form, the most common medicines contain diclofenac in the form of sodium or potassium salt (DFNa and DFK, respectively). However, because of the low $pK_{a}$ value one can expect that in living organisms the dynamic equilibrium would be established between the acidic and dissociated forms: $DFH+H_{2}O⇋DF^{-}+H_{3}O^{+}$. For this reason, in the present study both DFH and DF${}^{-}$ as well as DFNa are included into considerations and compared from the structural and electronic point of view.

The latest sparse theoretical reports suggest that chitosan interacts with diclofenac via covalent bonds [31] and the evidence comes from the spectrofluorimetric measurements. Still, the hypothetical structure of an adduct presented there is not obvious. Thus, more involved investigation is crucial. Moreover, the magnetic chitosan has been shown experimentally to feature high absorption affinity with drugs containing the carboxyl groups such as carbamazepine or diclofenac, highly benefiting from strong electrostatic interactions [14]. Therefore, the aim of the present study is to carefully analyze the intermolecular interactions between chitosan and exemplary NSAID (diclofenac) with computational chemistry tools. Since the size of the real chitosan polymeric chains is prohibitively large for reliable and in-depth theoretical calculations, three models are applied. For the simplest analysis, single chitosan unit is applied as the biomolecule interacting with diclofenac. Next, the fully-deacetylated chain of five units is considered. Additionally, the three chitosan chain modifications are studied with the five-unit models substituted according to out previous experimental investigation [18,27,29]. The strength of the mutual chitosan:drug interaction is analyzed with respect to the chosen model in order to determine the preferential structures and dominant interaction energy components. The determined computational tendencies remain in the agreement with our previous experimental data and allow for the explanation of the non-monotonic trends for modified chitosan chains.

It needs to be underlined that the present study concerns only the gas phase chitosan:diclofenac interactions. However one should realize, that in general, solvent presence can strongly affect the geometry of the investigated systems, their energetics, electronic properties and—what is of great importance—the characteristics of the created hydrogen bonds between the subunits of the investigated molecular complexes [32,33,34,35,36,37]. The numerous evidences of the modification of molecular spectroscopic properties, NMR chemical shifts, weak intra- and intermolecular interactions or proton transfer processes can be found for instance in older as well as more recent works by Sobczyk et al. and Limbach et al. [37,38,39,40,41,42,43]. Therefore, it can be crucial to include the solvent effects in computational studies in order to obtain quantitative correspondence to the experimental results. Intuitively, the conceptually simplest approach is to include explicitly solvent molecules around the solute. However, for the complete description of the behavior of the species of interest, usually large number of solvent molecules is necessary, what complicates the calculations significantly and often increases prohibitively their costs. Therefore, their practical realization usually boils down either to quantum calculations for relatively small solvation clusters or classical simulations in a box full of solvent. The combination of these two ways result in QM/MM approaches which can offer an interesting alternative with respect to the cost versus quality balance. Nevertheless, one need to take a particular care about the proper sampling of the solvent configuration space. On the other hand, so called implicit approaches have gained enthusiasm, because modeling a solvent as a continuous medium of a defined dielectric constant does not lead to a significant escalation of the required computational resources. However, this is possible at the expense of neglecting of the inclusion of the specific solute-solvent interactions in the conventional polarizable continuum models (PCM), thus causing problems with a proper description of weak non-covalent interactions mainly in polar protic solvents [43,44,45]. The improvement for the implicit solvent treatment, based on the charge density of the solute, has been introduced by Marenich et al. [46]. Their SMD model brings in not only the long-range bulk electrostatic polarization effects, but also to some extent short-range contributions to the free energy of solvation, arising from solute–solvent interactions in first solvation shell. This concept is claimed to allow the universal application of the SMD model for any solvents and any (both charged and uncharged) solutes, yet it was shown that the original SMD parametrization can be not optimal [47,48,49]. Among the new developments of environment treatment, one can mention the Adduct-under-Field (AuF) approach proposed by Shenderovich and Denisov [50,51,52] where environment effects are simulated by the presence of the fictitious external electric field.

In order to properly describe the quantitative character of the chitosan:diclofenac interactions, assuming the significant role of the hydrogen bonds in the complex stabilization, one needs to include the presence of the solvent molecules in calculations. However, for the above mentioned disadvantages of the implicit approaches, we decided to divide the current project into two parts. The first part, including the qualitative quantum mechanical gas phase study is presented here, while the advanced molecular dynamics study of chitosan chains interacting with diclofenac in water environment is currently in progress and its results will be published elsewhere.

## 2. Results

### 2.1. Diclofenac Interaction with Single Pristine Chitosan Unit

The initial study includes the investigation of the single chitosan unit (fully deacetylated six-membered heterocyclic system) interacting with a single diclofenac molecule. As the simplest model this approach cannot reproduce correctly all the interactions present in the real system with multiple long chitosan chains, however it would reveal the probable mutual orientation of the chitosan side groups and diclofenac thus giving rise to the initial structures for longer chitosan fragments.

**Figure 1:**
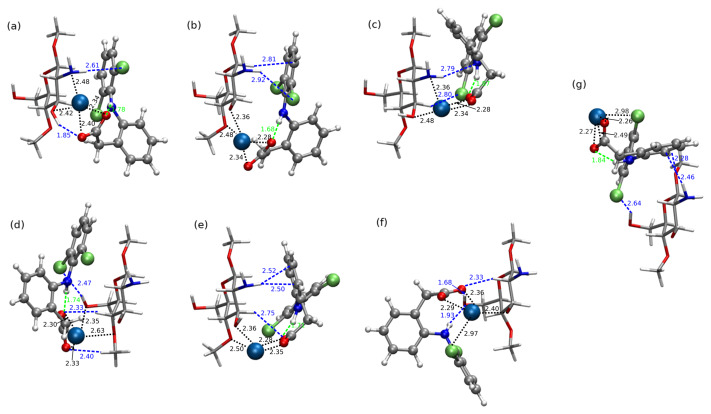
B97-D3/6-311++G(d,p) optimized structures of chitozan unit interacting with DFNa (explicit given the distances between the sodium cation and four coordinated atoms in Å—black color, and hydrogen–hydrogen-bond-acceptor distances—intramolecular in green and intermolecular in blue). The strongest attraction: (**a**) CS1:DFNa**4**, (**b**) CS1:DFNa**1**, (**c**) CS1:DFNa**6**, (**d**) CS1:DFNa**5**, (**e**) CS1:DFNa**9**, (**f**) CS1:DFNa**7a** and weakest attraction (**g**) CS1:DFNa**10**. Sodium cation depicted as large skye blue ball, DFNa in Corey–Pauling–Koltun representation and CS1—in licorice with following atom colors: chlorine—lime green, oxygen—red, nitrogen—cobalt blue, carbon—silver, hydrogen—white.

**Figure 2:**
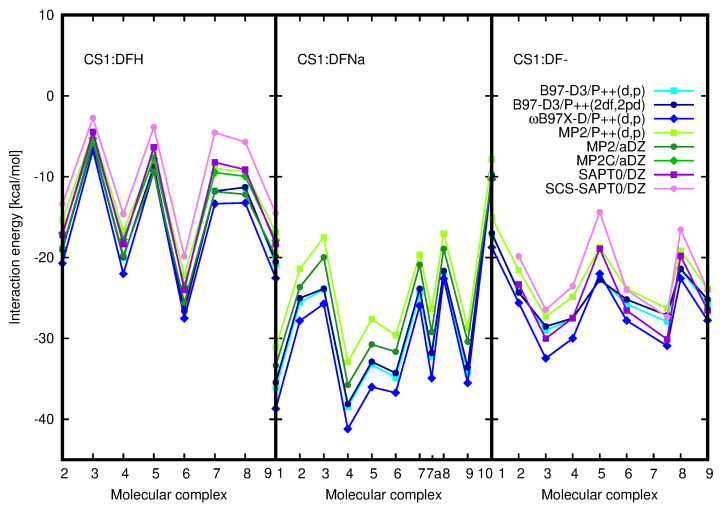
Supermolecular counterpoise corrected DFT (blue shades) and MP2 (green shades) and total SAPT0 and SCS-SAPT0 (violet shades) interaction energies for CS1:DFH, CS1:DFNa and CS1:DF${}^{-}$ complexes. MP2C results for CS1:DFH complexes (green diamonds) given for verification of the applied approaches. For basis set symbols see a caption for Table 1. Corresponding structures depicted in Supplementary Materials.

Exemplary structures were optimized with the B97-D3/6-311++G(d,p) approach and next counterpoise-corrected interaction energy calculated with both B97-D3 and long-range corrected ωB97X-D functional and the same triple-zeta basis set for all three analyzed diclofenac forms (DFNa, DFH and DF${}^{-}$). Additionally, the supermolecular interaction energy was also estimated with MP2 approach. The most stable CS1:DFNa complexes are depicted in Figure 1, and all the optimized CS1:DFNa structures are attached in Supplementary Information. The obtained interaction energies are presented in Table 1 and in comparison with other diclofenac forms depicted as a middle panel in Figure 2. First look at the obtained interaction energies allows to notice a general convergence of the supermolecular interaction energies with respect to the applied approach. Moreover, also the convergence with respect to a basis set has been tested and the acquired data are presented in Supporting Information. The inclusion of the larger set of diffuse functions is of marginal importance for a present qualitative considerations, therefore a triple-zeta quality basis set augmented with a conventional set of diffusion functions is applied further on.

It can be observed that except structures **5** and **9** for DFNa, the energetic order is retained for both applied functionals and second order of perturbation theory. The long-range corrected interaction energies are slightly more attractive, while MP2 underestimates the mutual attraction between the saccharide unit and the drug.

In the case of CS1:DFNa complexes, the most stable supermolecular interaction is observed in system **4**, where the diclofenac chlorinated ring is placed above the chitosan unit in a parallel manner. The diclofenac carboxide group is also turned into the direction of the chitosan unit, with the sodium cation coordinated by the two oxygen atoms from diclofenac carboxide group (2.34 Å and 2.40 Å) as well as the hydroxyl oxygen (2.42 Å) and amino nitrogen (2.48 Å) from chitosan unit. The additional stabilization of the system is acquired by the intermolecular hydrogen bond of the weaker type: N–H...Cl (2.61 Å between chlorine and hydrogen atoms, 3.63 Å between chlorine and nitrogen atoms and the N–H–Cl angle 171.6${}^{\circ }$). Furthermore, featuring from the weak C–H...C and C–H...N interactions, CS1:DFNa**4** is hold by a mutual attraction of the order of 40 kcal/mol (–38.46 kcal/mol for B97-D3/6-311++G(d,p) and –41.20 kcal/mol for ωB97X-D functional).

The next group of the CS1:DFNa complexes contains two pairs of similar structures: **6** and **9**, and **5** and **7a**, characterized by the B97-D3 interaction energies between –35 and –32 kcal/mol. In the first pair, the chlorinated ring is placed above the chitosan ring, while the ring with a carboxide group is shifted to the side of a chitosan amino group, in this manner maximizing the hydrogen bond N–H...N interaction in **6** and benefiting from N–H...C interactions in **9**. On the other hand, the second pair encloses the chlorinated ring below the chitosan unit and the carboxide group prone to the C–H...O and—in **7a** even O–H...O attraction, additionally to the O...H–N intercourse. The similar contact pattern is also observed in complex **8** of the B97-D3 interaction energy equal to –21.79 kcal/mol, however here the carboxide group is pushed aside and does not interact directly with CS1.

It has been previously shown by quantum dynamic tools that the great stabilizing role in acidic form of diclofenac is played by the intramolecular N–H...Cl interaction [53,54]. One of the local minima analyzed there correspond to the present DFH**4** structure. Despite the interaction with chitosan unit, here also diclofenac sodium preserves the strong intramolecular hydrogen O...H–N bond (1.78 Å, 2.72 Å and 27.7${}^{\circ }$, respectively). This intramolecular hydrogen bond is present in most of the optimized CS1:DFNa complexes except the complex **2** and its length is retained at the same order of magnitude (distances between hydrogen and hydrogen-bond-acceptor varying from 1.68 to 1.93 Å). The non-covalent interactions depicted with NCIPLOT in Figure 5 prove the presence of the weak intermolecular interactions between the complex units of both van der Waals type and hydrogen bonds.

The second strongest interaction is estimated for CS1:DFNa**1** complex, that closely resembles CS1:DFNa**4** with the difference between them being the rotation of DFNa in the plane of chlorinated ring. Thus, the sodium cation shifts from the area between amino and hydroxyl groups in CS1 to a space between the hydroxyl and methoxy chitosan substituents. The weak hydrogen N–H...Cl bonds are absent here and this weakens the material:drug interaction by about 2 kcal/mol with respect to the most attractive complex, giving here –36.10 kcal/mol and –38.68 kcal/mol for B97-D3 and ωB97X-D, respectively.

It should be also noticed that the presence of sodium cation between CS1 and diclofenac increases the mutual attraction of the two subsystems, while the placement of Na${}^{+}$ on the side, as in structure **10**, causes the meaningful reduction of the mutual interaction.

**Figure 3:**
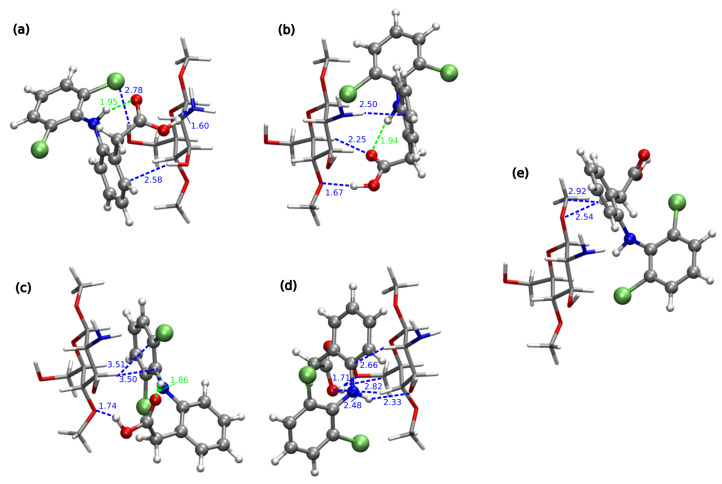
B97-D3/6-311++G(d,p) optimized most stable structures of chitozan unit interacting with DFH (explicite given the hydrogen–hydrogen-bond-acceptor distances and some other important contacts; blue for intermolecular contacts and green—for intramolecular): (**a**) CS1:DFH**6**, (**b**) CS1:DFH**9**, (**c**) CS1:DFH**4**, (**d**) CS1:DFH**2** and one with the weakest analyzed mutual attraction: (**e**) CS1:DFH**3**. DFH presented in Corey–Pauling–Koltun (CPK) representation and CS1—in licorice with following atom colors: chlorine—lime green, oxygen—red, nitrogen—cobalt blue, carbon—silver, hydrogen—white.

In comparison to CS1:DFNa complexes, the absolute values of interaction energies for CS1:DFH (chitosan unit with acidic form of diclofenac, see Figure 3) are smaller and the difference between the most favorable attraction and the weakest complex is equal to 29.49 kcal/mol for B97-D3/6-311++G(d,p) approach, while for CS1:DFNa the corresponding value accounts for 28.72 kcal/mol. The most beneficial attraction of −26.48 kcal/mol is noticed in complex CS1:DFH**6**, stabilized by the strong intermolecular hydrogen bond involving amino nitrogen from chitosan unit and the hydroxyl moiety from carboxyl group of diclofenac (N...H distance 1.60 Å, N...O distance 2.63 Å and N...H–O angle 164.5${}^{\circ }$). Another stabilizing interaction comes from O–H...Cl contact (2.79 Å, 3.73 Å and 162.9${}^{\circ }$, respectively for a hydrogen– acceptor distance, donor–acceptor distance and a valence angle donor–hydrogen–acceptor) and weaker O–H...C and C–H...C contacts. In addition, here the intramolecular hydrogen N–H...O bond is present in a diclofenac acid.

Next in line is the group of three complexes characterized by the interaction energies between −21 and −19 kcal/mol, namely: CS1:DFH**9** (−20.77 kcal/mol), CS1:DFH**4** (−20.06 kcal/mol) and CS1:DFH**2** (−19.04 kcal/mol ). In all of them the crucial stabilizing role can be ascribed to O...H–O intramolecular interactions. Furthermore, several weaker N–H...C and C–H...C contacts are present in each of these complexes. Structure CS1:DFH**2** benefits additionally from the O...H–N intermolecular interaction, however its stability is damped by the lack of the intramolecular O...H–N hydrogen bond in diclofenac acid. This occurs also in CS1:DFH**3**, the weakest of all analyzed CS1:DFH complexes and seems to be not counterbalanced by other intermolecular interactions, giving the interaction energy of only −5.99 kcal/mol. In the complexes with moderately small interaction (−12 to −11 kcal/mol, namely CS1:DFH**7** and CS1:DFH**8**), only weaker C–H...N or O...H–C contacts are present and no intermolecular strong hydrogen bond can be observed. These observations are also confirmed by the non-covalent interaction analysis presented in Figure 6.

The quality of the present results for DFT and conventional MP2 approach is confirmed additionally for CS1:DFH complexes with the MP2C method that provides an improved long-range correlation with respect to the traditional perturbation theory. The obtained supermolecular interaction energy values closely resemble those for MP2 and SCS-SAPT0. Therefore, one can assume also the qualitative propriety of the dispersion component even in the DFT interaction energies for the remaining systems and therefore the B97-D3 functional will be applied for the large models of substituted chitosan chains and chitosan dimers.

**Figure 4:**
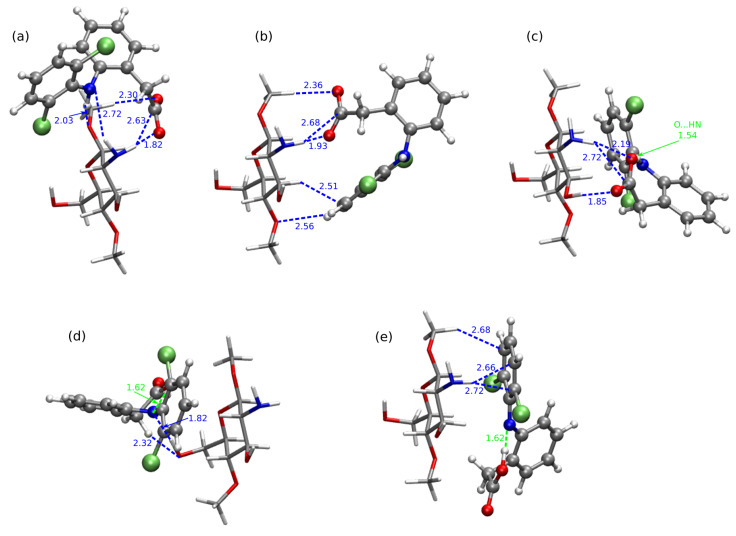
B97-D3/6-311++G(d,p) optimized most structures of chitozan unit interacting with DF${}^{-}$ with corresponding interaction energies, (**a**) CS1:DF${}^{-}$**3**, (**b**) CS1:DF${}^{-}$**7a**, (**c**) CS1:DF${}^{-}$**4**, and two least stable structures exhibiting a intramolecular proton transfer in DF${}^{-}$ moiety, (**d**) CS1:DF${}^{-}$**5-PT** and (**e**) CS1:DF${}^{-}$**1-PT** (explicit given the distances of the hydrogen–hydrogen-bond-acceptor distances in Å: green for intramolecular interaction and blue for intermolecular interaction). DF${}^{-}$ presented in Corey–Pauling–Koltun (CPK) representation and CS1—in licorice with following atom colors: chlorine—lime green, oxygen—red, nitrogen—cobalt blue, carbon—silver, hydrogen—white. PT denotes the proton transfer.

**Figure 5:**
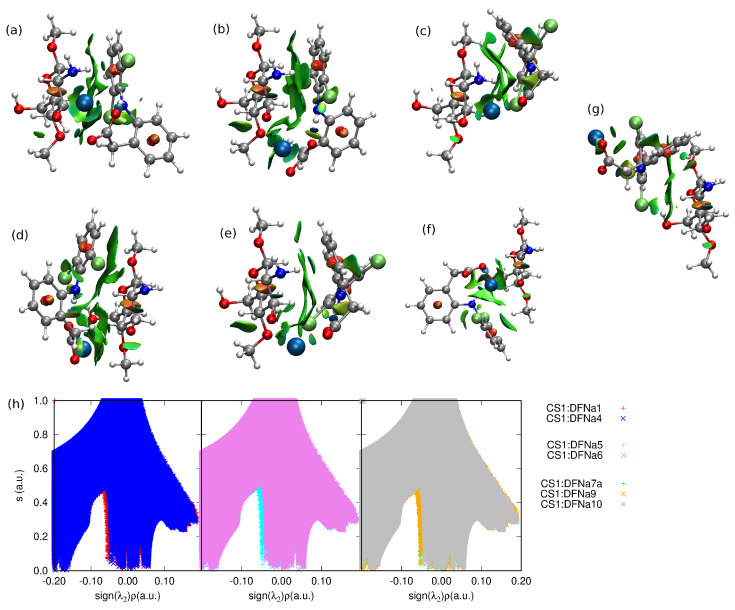
NCIPLOT of the most stable structures of chitozan unit interacting with DFNa: (**a**) CS1:DFNa**4**, (**b**) CS1:DFNa**1**, (**c**) CS1:DFNa**6**, (**d**) CS1:DFNa**5**, (**e**) CS1:DFNa**9**, (**f**) CS1:DFNa**7a**, the weakest attraction (**g**) CS1:DFNa**10** and (**h**) the NCI relation between the reduced gradient, *s* and the sign of the second eigenvalue, $\lambda_{2}$, of the electron-density Hessian. Atom colors: chlorine—lime green, oxygen—red, nitrogen—cobalt blue, carbon—silver, hydrogen—white.

**Figure 6:**
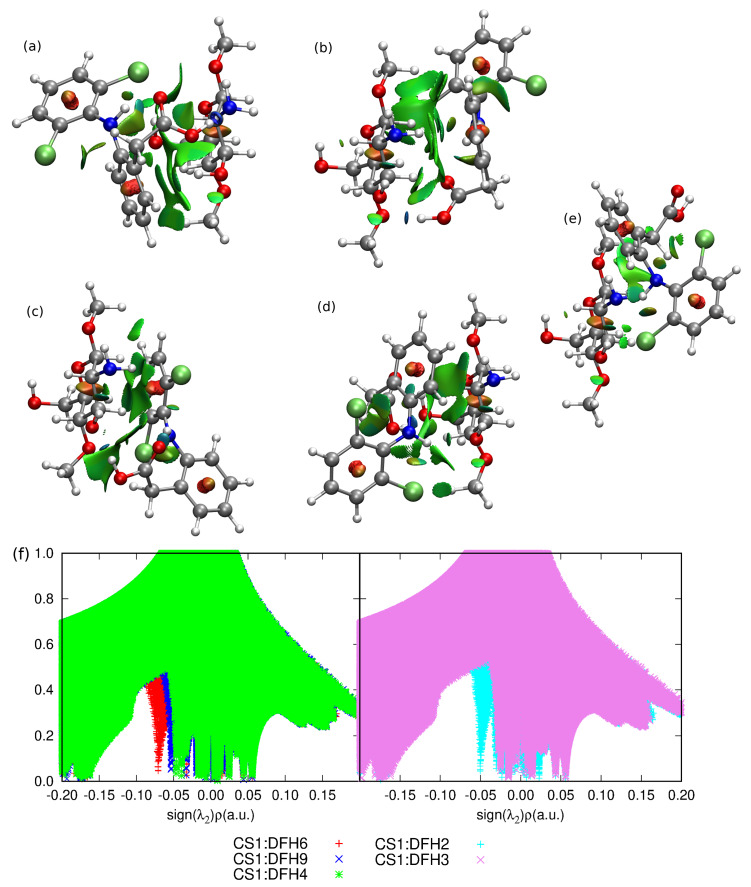
NCIPLOT of the most stable structures of chitozan unit interacting with DFH: (**a**) CS1:DFH**6**, (**b**) CS1:DFH**9**, (**c**) CS1:DFH**4**, (**d**) CS1:DFH**2** and one with the weakest analyzed mutual attraction: (**e**) CS1:DFH**3**, (**f**) the NCI relation between the reduced gradient, *s* and the sign of the second eigenvalue, $\lambda_{2}$, of the electron-density Hessian. Atom colors: chlorine—lime green, oxygen—red, nitrogen—cobalt blue, carbon—silver, hydrogen—white.

The importance of the intramolecular hydrogen bonds for complex stabilization seems to be slightly different for the diclofenac anion interaction. Among the optimized stable structures of CS1:DF${}^{-}$ presented in Figure 4, the two most attractive complexes **3** and **7a** are void of intramolecular N–H...O hydrogen bond. The –N–H and –COO${}^{-}$ groups are pushed aside from each other, thus giving priority to the intermolecular interactions. However, the significance of the N...H...O intramolecular motif in DF${}^{-}$ can be noticed in **1** and **5** complexes, where the strong electrostatic attraction in this part of a system leads to the proton transfer from the amino group to the carboxide group (the complexes CS1:DF${}^{-}$**1** and CS1:DF${}^{-}$**5** will be presented further on in tables and figures and where necessary, denoted with the PT acronym standing for proton transfer).

**Figure 7:**
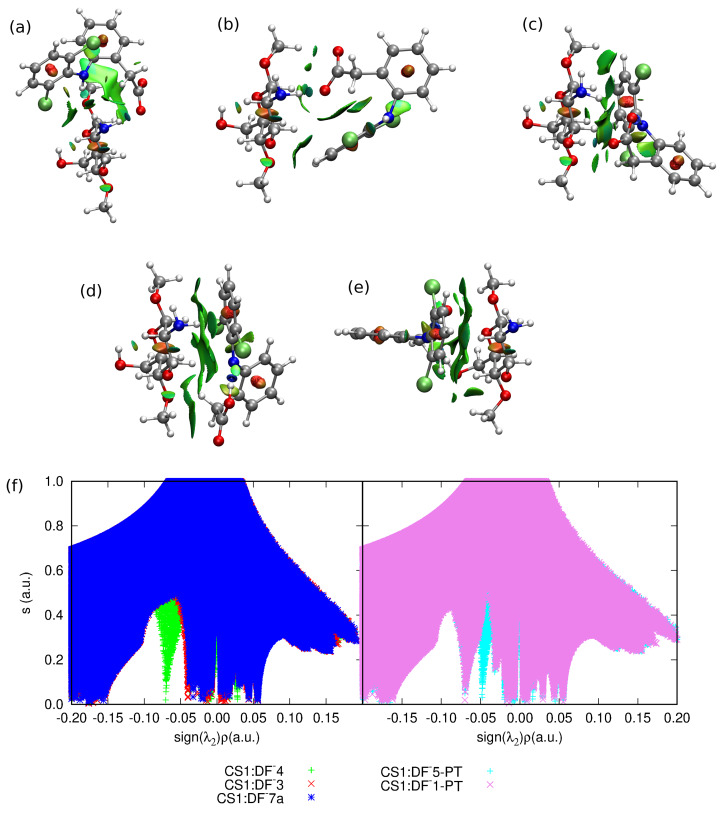
NCIPLOT of the most stable structures of chitozan unit interacting with DF${}^{-}$: (**a**) CS1:DF${}^{-}$**3**, (**b**) CS1:DF${}^{-}$**7a**, (**c**) CS1:DF${}^{-}$**4**, the weakest attraction for proton-transfer structures: (**d**) CS1:DF${}^{-}$**5-PT**, (**e**) CS1:DF${}^{-}$**1-PT** and (**f**) the NCI relation between the reduced gradient, *s* and the sign of the second eigenvalue, $\lambda_{2}$, of the electron-density Hessian. Atom colors: chlorine—lime green, oxygen—red, nitrogen—cobalt blue, carbon—silver, hydrogen—white. PT denotes the proton transfer.

In the most stable anionic complex CS1:DF${}^{-}$**3**, the dominant attractive role can be ascribed to the chitosan amino group involved in strong hydrogen bonds to amino and carboxide groups of DF${}^{-}$ (compare Figure 7). Here diclofenac approaches from the side, thus minimizing the aromatic ring interactions with CS1, and exposing its functional groups for mutual attraction.

Taking into account that the initial structures chosen for optimization arise from the same starting point for all the forms: DFH, DFNa and DF${}^{-}$, one can compare the similarities and differences in mutual interactions originating from the different protonation/ionization states of the drug. The clear difference can be noticed between structures **6** of CS1:DFNa and CS1:DFH, and similarly between **7a** of CS1:DFNa and CS1:DF${}^{-}$. In the first pair mentioned, the initial position of the drug is on the side of chitosan unit. The presence of sodium cation in a close proximity to the chitosan amino group and one of the hydroxyls keeps diclofenac carboxide group strongly bound and allowing only for the relaxation of the remaining part of the molecule in order to maximize the interaction of the chlorinated ring with the carbohydrate unit. On the other hand, in the case of the protonated diclofenac, the chlorine atom is bound by the hydrogen bond interaction with a chitosan unit, while the second aromatic ring of DFH shifts below CS1, exploiting the possible weak O–H...C and C–H...C contacts. Starting the geometry optimization of CS1:DFH**6** from the optimized CS1:DFNa**6** leads to the complex structurally similar to the initial architecture, however less stable than the proper CS1:DFH**6** by 6.22 kcal/mol. Similar reoptimization of the anionic complex CS1:DF${}^{-}$**7a** gives a complex of the geometry comparable to CS1:DFNa**7a**, with the energy higher by 1.01 kcal/mol than the original CS1:DF${}^{-}$**7a** complex.

#### Nature of the CS1: Diclofenac Interaction

**Figure 8:**
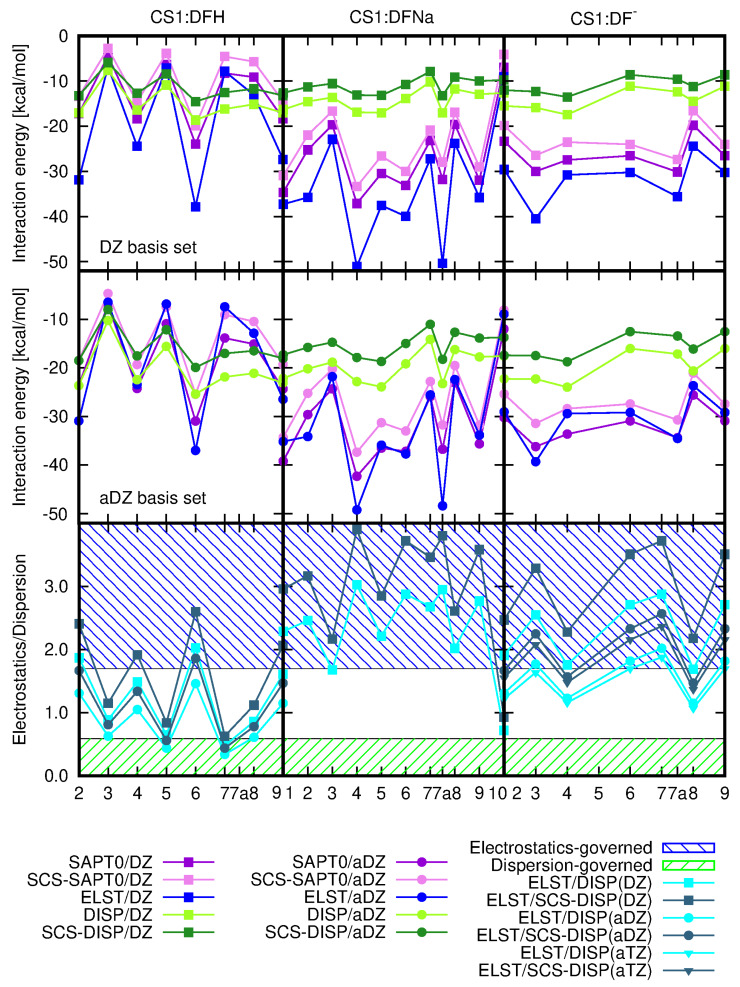
SAPT0 total interaction energy and electrostatic and dispersion components for investigated CS1:drug complexes and a relation between electrostatic and dispersion interaction.

SAPT0 procedure allows for the decomposition of the total interaction energy into components of the well-defined physical origin. The electrostatic and dispersion components together with total SAPT0 and SCS–SAPT0 interaction energies and the electrostatics–dispersion ratio for the investigated CS1:drug complexes are depicted in Figure 8 and their numerical values are given in Table 2. Additionally, the induction and exchange components are provided in Supplementary Materials. The recommendations advise to apply the aug-cc-pVDZ basis set in order to obtain the well-balanced results with the proper saturation of the correlation and basis set effects, that benefit from mutual error cancellation [55,56,57,58,59]. However, here one can see that even the smallest applied cc-pVDZ basis set keeps a good qualitative agreement with general tendencies, similarly as in the case of supermolecular calculations.

The careful investigation of the electrostatic and dispersion components and the total interaction energy shows that in the case of DFH interacting with chitosan unit the energetic order of the complexes remains almost independent on the applied approach going from supermolecular (with 6-311++G(d,p) basis set) to perturbative treatments and separate electrostatic and dispersion contributions with DZ basis set. The eventual interchange of the structures in the energetic series arise from the tiny modifications in interaction energies. The parallel character of the dispersion and electrostatic components should be underlined here, since in the two other types of complexes the total SAPT0 interaction energy follows closely electrostatic contribution, however the sequence for the dispersion component values does differ significantly. On the other hand, the plot of the dispersion energy component for all the investigated species is much flatter than electrostatic plot, exhibiting large variation of the component arising from Coulomb interactions between partial charges. The dispersion energy component remains crucial particularly for the stabilization of CS1:DFH complexes, where the lack of electron correlation effects produces repulsive interaction at the HF level of theory in as many as four systems. This arises from the large positive exchange component exceeding the absolute value of electrostatics. In the adsorption of DFNa and DF${}^{-}$ on chitosan unit, the most pronounced effect of dispersion interactions is noticed in CS1:DFNa**10**, and for the rest of the complexes the exchange component is counterbalanced by the electrostatic interaction and the total HF interaction energy can be attractive.

The comparison of the electrostatic–dispersion ratio, presented in Figure 8 on the lowest panels and in Table 2, indicates the difference in the nature of the investigated complexes. Considering complexes with electrostatic–dispersion ratio larger than 1.7 as electrostatics-governed and such with the ratio smaller than 0.59 as dispersion-governed, according to Rezač et al. [60], allows to classify the CS1:DFH complexes as mixed with non-negligible dispersion, CS1:DFNa—as mostly electrostatic and CS1:DF${}^{-}$ as balanced mixed. One can also notice that the increase of the basis set leads to the increasing significance of dispersion forces, however this effect seems to be almost saturated for the aug-cc-pVTZ basis set. The opposite impact is seen for the inclusion of the spin component scaling: the SCS–dispersion contribution is slightly diminished with respect to the dispersion with no spin distinction (compare the circles depicting the ratios of electrostatics to SCS–dispersion and squares depicting the corresponding ratio with no-SCS–dispersion in lowest panels of Figure 8).

The attempts of finding clear correlations between the complex structure and interaction energy components are doomed to failure due to the large number of degrees of freedom. However, some relations can be observed. Namely, in the case of CS1:DFNa, complex **10** is characterized by the mixed character, yet with the large importance of dispersion component (see Table 2). This tendency is confirmed by the larger values of the dispersion and SCS-dispersion components than the electrostatic energy (electrostatic–dispersion ratio smaller than 1). Comparison of these data with the structural arrangements points out the significance of the presence of the sodium cation between the diclofenac moiety and a chitosan unit for the electrostatic nature of this interaction. If, as in CS1:DFNa**10**, sodium is shifted out to the side (compare Figure 1), prohibiting its direct contact with chitosan side groups, the dispersion forces get relevant.

### 2.2. Diclofenac Interaction with Pristine Five-Unit Chitosan Chain

**Figure 9:**
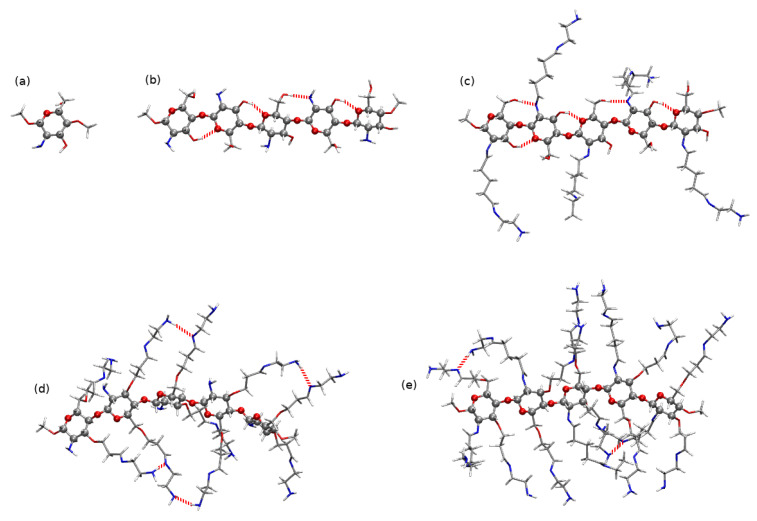
Investigated models of chitosan: (**a**) pristine single chitosan unit CS1, (**b**) pristine five-units chain fragment CS5 and substituted five-units chain fragments: (**c**) single –NH${}_{2}$-substituted CS5(NH${}_{2}$), (**d**) double –OH-substituted CS5(NH${}_{2}$)${}_{2}$ and (**e**) all three functional groups-substituted CS5(NH${}_{2}$)${}_{3}$. Ball representation used for atoms in the main chain and licorice for side chains. Atom colors: red—oxygen, blue—nitrogen, silver—carbon, white—hydrogen. Red dashed lines sketch intramolecular hydrogen bonds according to the following criteria: distance between the hydrogen bond donor and hydrogen bond acceptor equal to 3.5 Å and the angle H–donor–acceptor 30${}^{\circ }$.

Fully deacetylated chains are taken here as the example of the chitosan chain (compare Supporting Information, Figure S6). Of the four initial structures presented in Supplementary Materials differing with the rotation of the side functional groups, for the further study the one with the lowest energy has been chosen (panel (b) in Figure 9). One can notice that all the investigated chains are not planar and exhibit some symptoms of helixity. Moreover, in all of them strong intramolecular hydrogen bonds are present between the subsequent saccharide units. A linear chain creates strong intramolecular hydrogen bonds of the type O3–H...O5 between the hydroxyl groups in the *n*-th unit and the oxygen in the ring of the n+1-th unit and N–H...O and O–H...N between the amino- and hydroxyl side groups in subsequent units. These interactions between the CS units allows to retain the chain stiffness and prevents its uncontrollable winding into the helical structure for such a short chain fragment. Nevertheless, one need to remember that in the case of the long oligo- and polysaccharide chains the 2-fold helical conformation is confirmed by both experimental techniques and molecular dynamics study [1,2].

**Figure 10:**
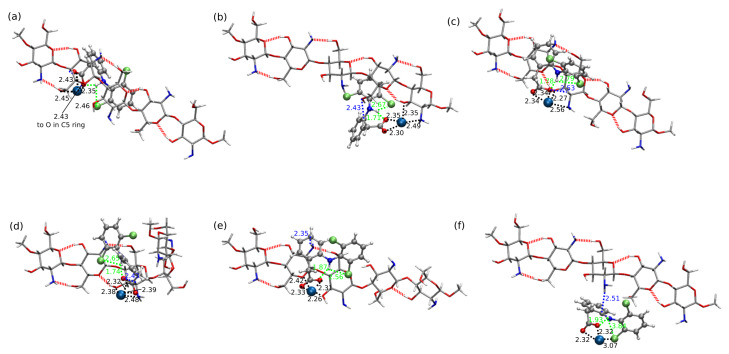
B97-D3/6-311++G(d,p) interaction in most attractive: (**a**) CS5:DFNa**8**, (**b**) CS5:DFNa**6**, (**c**) CS5:DFNa**10**, (**d**) CS5:DFNa**7**, (**e**) CS5:DFNa**2** and the weakest of all the analyzed CS5:DFNa complexes: (**f**) CS5:DFNa**3**. Blue dashed lines and labels present intramolecular hydrogen bond in diclofenac sodium, green dashed lines and labels: intermolecular hydrogen bonds, red dashed lines depict intramolecular hydrogen bonds in chitosan chain and black lines and labels: distances from sodium cation to its nearest neighbors. This convention is used further on.

**Figure 11:**
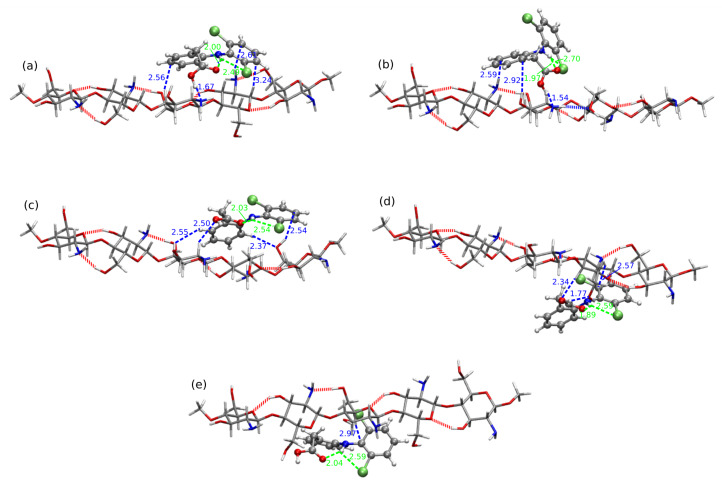
B97-D3/6-311++G(d,p) interaction in most attractive: (**a**) CS5:DFH**1**, (**b**) CS5:DFH**10**, (**c**) CS5:DFH**5**, (**d**) CS5:DFH**9** and the weakest CS5:DFH complexes (**e**) CS5:DFH**8**.

**Figure 12:**
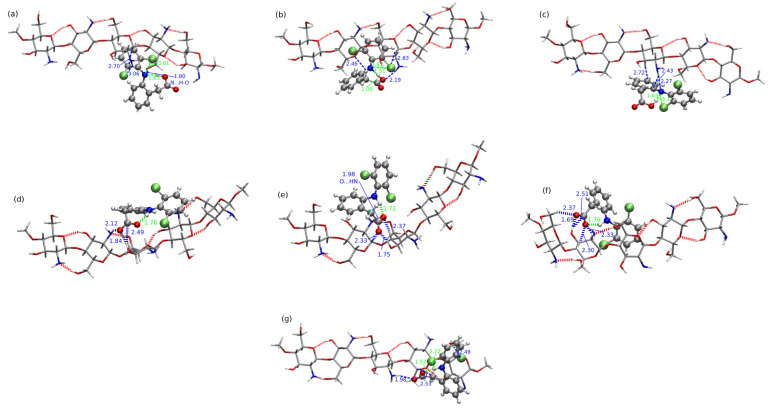
B97-D3/6-311++G(d,p) interaction in most attractive and the weakest CS5:DF${}^{-}$ complexes (note the proton transfer, PT, occuring in diclofenac for the most attractive complexes): (**a**) CS5:DF${}^{-}$**6-PT**, (**b**) CS5:DF${}^{-}$**4-PT**, (**c**) CS5:DF${}^{-}$**3-PT**, (**d**) CS5:DF${}^{-}$**1**, (**e**,**d**) CS5:DF${}^{-}$**2**, (**f**,**d**) CS5:DF${}^{-}$**8** and (**g**,**d**) CS5:DF${}^{-}$**7**.

**Figure 13:**
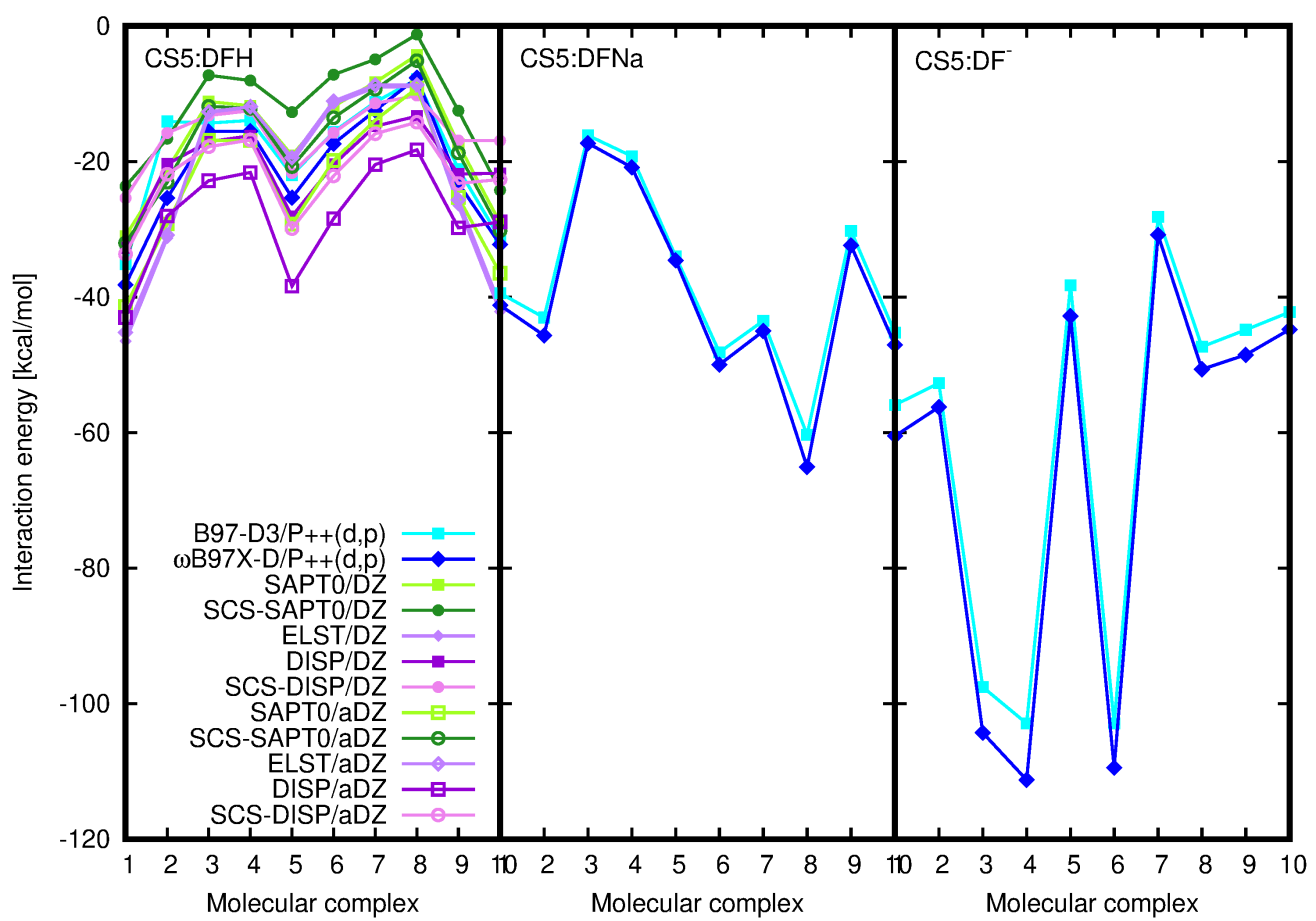
Counterpoise-corrected supermolecular B97-D3/6-311++G(d,p) and ωB97X-D/6-311++G(d,p) supermolecular interaction energy (blue lines) compared with SAPT0 results (green lines) and interaction energy components (violet lines) for CS5:DFH system and supermolecular data for CS5:DFNa and CS5:DF${}^{-}$ complexes.

Figure 10, Figure 11 and Figure 12 depict the lowest-energy complexes of CS5 with all forms of diclofenac (all the analyzed geometries are presented in Supporting Information). Corresponding supermolecular interaction energies and SAPT0 total energies are plotted in Figure 13. The general tendency for CS5:DFNa is similar as in the case of CS1:DFNa complexes: the cation directed to the side of the system and not interacting directly with any part of chitosan leads to the least stable arrangement. On the other hand, the stabilization of the mutual interaction in the most attractive system CS5:DFNa**8** is caused by the interaction of the cation with the oxygens of the –OH groups in CS5 and additional hydrogen bond present between –COO${}^{-}$ of diclofenac sodium and a C-H moiety of chitosan chain. Additionally, the intramolecular hydrogen bonds are also present both in chitosan chain and in DFNa subsystem. The supermolecular B97-D3/6-311++G(d,p) interaction energy for this complex is equal to −60.35 kcal/mol and the atrraction is by 12.21 kcal/mol stronger than in the next localized minimum, namely CS5:DFNa**6**. Here, additionally to the sodium cation caught between amino nitrogen and hydroxyl oxygen of chitosan chain, two intermolecular hydrogen bonds are present: between hydroxyl oxygen in CS5 and C-H in diclofenac and the second one between –O–H of the same CS5 module and amino nitrogen in DFNa. The intramolecular hydrogen bond in diclofenac is also present in CS5:DFNa**6**. After the careful investigation of the presented CS5:DFNa structures, one can notice that the intramolecular –O...H–N interaction in diclofenac can form in all of the structures, however in the case of CS5:DFNa**3**, which is the weakest complex, the above mentioned contacts are significantly weaker (longest distances or more deformed from linear angles).

The mutual interaction between acidic form of diclofenac and CS5 is in general weaker than the CS5:DFNa interaction (compare blue curves in Figure 13). The strongest attraction is noticed for the complexes with intramolecular N–H...Cl hydrogen bond in DFH moiety as well as the strong intermolecular hydrogen bonds between DFH and CS5. However here the intramolecular hydrogen bond stabilizing diclofenac, becomes more deformed from linearity in numerous cases, patricularly in CS5:DFH**3**, where the N–H...O angle is equal to 137.5${}^{\circ }$. The most stable CS5:DFH structures possess the strong (and thus relatively short) hydrogen bond contacts between the complex fragments. Namely, in CS5:DFH**1**, the shortest distance is observed for (diclofenac)O–H...N(chitosan) contact and is equal to 1.67 Å. Both of these systems exhibit interaction much stronger than next in line (−35.19 kcal/mol and −31.41 kcal/mol, respectively). In the second most stable system, CS5:DFH**10**, the corresponding distance is even shorter (1.54 Å). Two next complexes with the interaction energy in the range of −22.00 kcal/mol to −21.00 kcal/mol adopt the arrangement that prevents the strong O–H...N intermolecular interaction. Thus, CS5:DFH**5** gets stabilization only from the weaker contacts such as C–H...O. On the other hand, the rotation of DFH with respect to the chitosan strand allow for the formation of the strong (diclofenac)O–H...O(chitosan) interaction of the length 1.77 Å. Additionally, the mutual arrangement of the carboxyl group in DFH and hydroxyl group in chitosan leads to the six-membered cyclic pattern built of (diclofenac)O${}^{-}$–C–O–H...O–H(chitosan) fragments.

In the case of CS5 interacting with diclofenac anion one can recognize the same susceptibility for the intramolecular proton transfer phenomenon as it was in the case of shorter CS1 moiety. Among the analyzed structures, the three of the most attractive interactions, CS5:DF${}^{-}$**6-PT** (−102.91 kcal/mol), CS5:DF${}^{-}$**4-PT** (−102.90 kcal/mol) and CS5:DF${}^{-}$**3-PT** (−97.53 kcal/mol), arise from proton transfer. Their interaction energy is almost twice as large as for the next complex, CS5:DF${}^{-}$**1** with the interaction −55.91 kcal/mol, that is not prone to proton transfer. In the structure with the weakest interaction, the short intramolecular hydrogen bond is present in an anion (1.57 Å) as well as the intermolecular contacts ((anion)O...H–N(chitosan) of 1.98 Åand ((anion)Cl...H–O(chitosan) of 2.49 Å). Thus, taking into account all the complexes with anion, one can erroneously assume that the interaction of CS5 with diclofenac anion can be much stronger than those of CS5 with diclofenac sodium. However, excluding the proton transfer structures, it occurs that the interaction energies of CS5:DF${}^{-}$ are of the same order of magnitude as CS5:DFNa.

Figure 13 beside the supermolecular interaction energy presents also the electrostatic and dispersion components as well as the total SAPT0 and SCS-SAPT0 interaction energy for CS5:DFH complexes. One can notice the similarities of all the lines depicted in CS5:DFH panel—the general supermolecular qualitative tendencies are reproduced by SAPT0 and both electrostatic and dispersion energy. The character of the mutual CS5:DFH interactions is for the most part mixed. In the case of three or four systems (depending on the approach applied) the electrostatic component slightly outweighs the dispersion (CS:DFH**1**, CS:DFH**2**, CS:DFH**9**, CS:DFH**10**) and in the remaining complexes dispersion is somewhat larger than electrostatics. On the basis of the criteria applied by Režac et al. [60] one can distinguish here only two complexes (SAPT0/aDZ) definitely governed by dispersion forces. The inclusion of spin-component scaling act in favor of dispersion character also in the CS:DFH**3**, CS:DFH**4**, CS:DFH**5** and CS:DFH**8** complexes.

### 2.3. Diclofenac Interaction with Substituted Chitosan Chain

Aside from the structural and energetic aspects of the interaction of a biopolymer with a selected NSAID, the subject of the present study is an analysis of the mutual interaction of a modified chitosan material with diclofenac that should verify the ability of its application in the waste water decontamination from the drug pollutions. As a result of the size of the previously analyzed systems as well as the general complication of the problem (deacetylation degree or the presence of the solvent included in MD simulations), here the focus is provided on the fully deacetylated five-units chains, substituted in the same manner in each unit, CS(NH${}_{2}$)${}_{x}$ with x=1,2,3. The analyzed low-energy conformations of modified chitosan chains are presented in Figure 9 (panels (c), (d) and (e), respectively for x=1,2,3).

**Figure 14:**
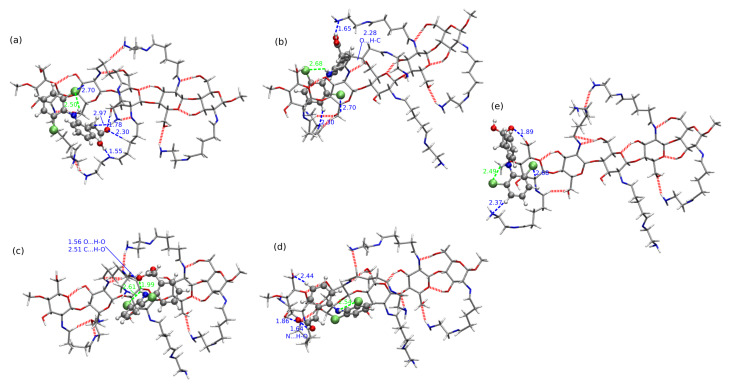
B97-D3/6-311++G(d,p) interaction in most attractive CS5(NH${}_{2}$):DFH complexes: (**a**) CS5(NH${}_{2}$):DFH**5**, (**b**) CS5(NH${}_{2}$):DFH**3**, (**c**) CS5(NH${}_{2}$):DFH**7**, (**d**) CS5(NH${}_{2}$):DFH**1** and the weakest complex: (**e**) CS5(NH${}_{2}$):DFH**2**.

Figure 14 shows the four CS5(NH${}_{2}$):DFH complexes with the most attractive interaction energy and the least stable system as panel (e) (all the complexes are presented in Supplementary Information). The strongest interaction is observed in the case of CS5(NH${}_{2}$):DFH**5** and is equal to −51.45 kcal/mol in the B97-D3/6-311++G(d,p) approach. Next three complexes presented in Figure 14, namely CS5(NH${}_{2}$):DFH**3**, CS5(NH${}_{2}$):DFH**7** and CS5(NH${}_{2}$):DFH**1**, exhibit the mutual attraction between the subsystems of the order of 40 kcal/mol (−41.04, −40.08 and −38.93 kcal/mol, respectively). On the other hand, in the least stable systems DFH and modified chitosan attract each other with the energy of −18.93 kcal/mol (CS5(NH${}_{2}$):DFH**2**) or −21.03 kcal/mol (CS5(NH${}_{2}$):DFH**13**). The remaining values of the interaction energy are given in Table 3.

For the complicated nature of the mutual interaction and numerous possible attractive contributions, it is extremely difficult to correlate the interaction energy with any other structural motifs present in the investigated complexes. However, one can see that due to the significant number of O–H...O bonds between the hydroxyl groups in the main chain and additional contribution from the H–bonds arising from the terminal amino groups in substituents, the polymer forms a planar, sheet-like structure allowing for the diclofenac approaching from each direction to the main saccharide chain. Since DFH has different comfortable ways of coming near chitosan, it can either find a favorable spot for its mutual attraction with saccharide rings or exploit the functional groups in the side chains. The side chains can entwine around the guest drug molecule, thus stabilizing the interaction. It can be seen for instance in CS5(NH${}_{2}$):DFH**5**, where three long substituents form cage-like, capturing the ring with –CH${}_{2}$COOH group. The strong intermolecular hydrogen bonds can be noticed both of the O–H...N and O–H...O type. In addition, the weaker interactions are present, such as C–H...N, C–H...Cl, C–H...O. It should be also noticed that the strong intramolecular O...N–H hydrogen bond in DFH is present in CS5(NH${}_{2}$):DFH**7** (Figure 14, panel (c)). In the remaining structures, the O...H–N contact vanishes due to the rotation of the carboxyl group in DFH in such a way to maximally exploit the electrostatic interactions with side functional groups in substituted chitosan chain. In several complexes (see Figure 14) the chlorinated ring adopts the position that allows to take advantage from intramolecular Cl...H–N interaction with the chlorine-hydrogen distance of the order of 2.5 Å or larger.

The chitosan with the hydroxyl groups in the main chain substituted with the long alkyl chains terminated with amino groups possesses the different rigidity and general shape than CS5(NH${}_{2}$):DFH. The net of the strong hydrogen bonds close to the polymeric core in CS5(NH${}_{2}$)${}_{2}$:DFH cannot form so easily as in the case of the chain with only one lenghty substituent, since the amino gropus remaining close to the chain are too distant from one another or from the terminal amino groups to form strong interactions. Only some weaker contacts of the N...H–C type are present close to the saccharide rings. Additionally, one can also notice the presence of the N–H...N hydrogen bonds arising between the terminal amino and imino groups in substituents, however their stability during the previous dynamical simulation has not been confirmed [27].

**Figure 15:**
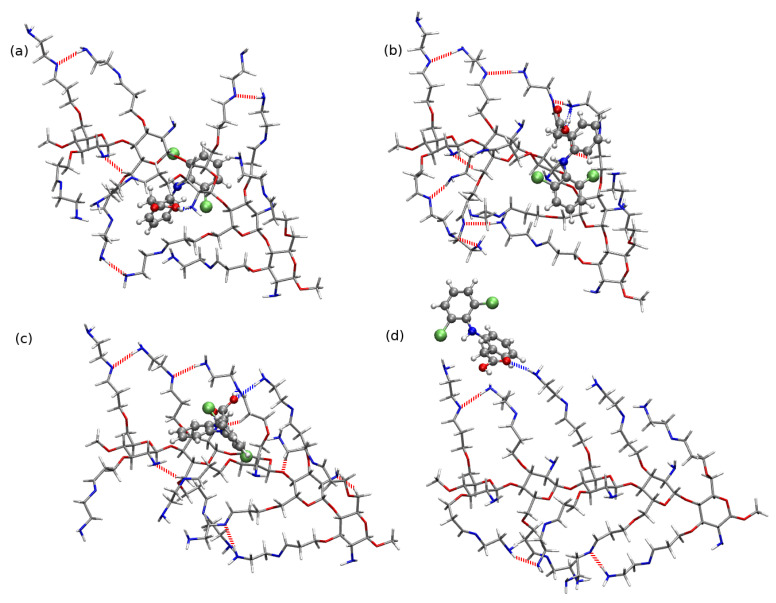
B97-D3/6-311++G(d,p) interaction in most attractive and the weakest CS5(NH${}_{2}$)${}_{2}$:DFH complexes: (**a**) CS5(NH${}_{2}$)${}_{2}$:DFH**14**, (**b**) CS5(NH${}_{2}$)${}_{2}$:DFH**10**, (**c**) CS5(NH${}_{2}$)${}_{2}$:DFH**8** and (**d**) CS5(NH${}_{2}$)${}_{2}$:DFH**5**.

The careful investigation of the most stable complexes exhibits the presence of strong O–H...N hydrogen bonds between the carboxyl group of DFH and amino groups of CS5(NH${}_{2}$)${}_{2}$ (either in the main chain or terminal ones). The conformational flexibility of diclofenac also in this case leads to the rotation of the –COOH group to the most favorable sites in the polymeric chain at the expense of the intramolecular O...H–N stabilization. In general, among all the analyzed CS5(NH${}_{2}$)${}_{2}$:DFH complexes, only four maintain the intramolecular N–H...O hydrogen bond, namely CS5(NH${}_{2}$)${}_{2}$:DFH**13**, CS5(NH${}_{2}$)${}_{2}$:DFH**6**, CS5(NH${}_{2}$)${}_{2}$:DFH**12**, CS5(NH${}_{2}$)${}_{2}$:DFH**2** (with the corresponding interaction energies equal to −36.81, −34.08, −31.55 and −19.64 kcal/mol, respectively). In all the strongly interacting structures presented in Figure 15, one can notice that the lengthy side chains form a shallow “quasi-bowl”, softly inclosing the drug. The first symptoms of such a prefered arrangement have been already seen in CS5(NH${}_{2}$):DFH complexes, however here this tendency is more pronounced. Unlike, in the least stable complex CS5(NH${}_{2}$)${}_{2}$:DFH**5** (Figure 15, panel (d)) DFH is shifted out from the “bowl” and constitutes only single O...H–N contact fulfilling the adopted in the present work (3.5 Å distance and 30${}^{\circ }$ angle). The remaining part of DFH here does not enter any other strong contacts except several possible junctions including C–H bonds.

The strength of the mutual diclofenac:modified chitosan interaction is of similar order of magnitude for the two lengthy substituents in polymeric chain as is was observed for CS5(NH${}_{2}$). The least stable minimum of CS5(NH${}_{2}$)${}_{2}$ found in the present study is characterized by the mutual attraction of −19.64 kcal/mol, while the strongest attraction noticed for CS5(NH${}_{2}$)${}_{2}$:DFH**14** is equal to −44.26 kcal/mol (compare Table 3). Similar DFH placement also is observed in the case of the second least attractive system, namely CS5(NH${}_{2}$)${}_{2}$:DFH**4**.

**Figure 16:**
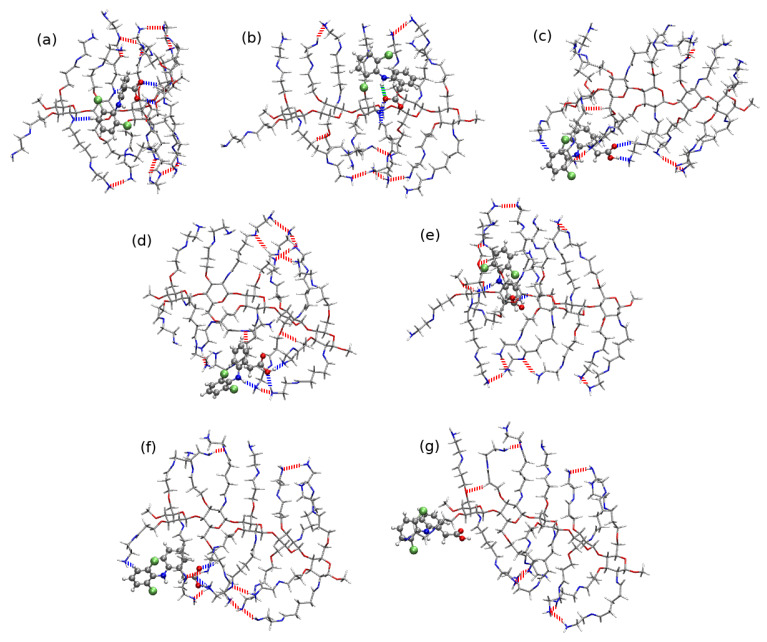
B97-D3/6-311++G(d,p) interaction in most attractive structures of CS5(NH${}_{2}$)${}_{3}$:DFH, namely: (**a**) CS5(NH${}_{2}$)${}_{3}$:DFH**10**, (**b**) CS5(NH${}_{2}$)${}_{3}$:DFH**20**, (**c**) CS5(NH${}_{2}$)${}_{3}$:DFH**12**, (**d**) CS5(NH${}_{2}$)${}_{3}$:DFH**11**, (**e**) CS5(NH${}_{2}$)${}_{3}$:DFH**18**, (**f**) CS5(NH${}_{2}$)${}_{3}$:DFH**13-PT** and the weakest complex, (**g**) CS5(NH${}_{2}$)${}_{3}$:DFH**14**

The largest analyzed derivative of chitosan, substituted with three lengthy alkyl chains terminated with amino groups exhibit more noticeable rigidity and from outside can be perceived as a sheet rather than a bowl, as it was in the case of CS5(NH${}_{2}$)${}_{2}$:DFH complexes. The heavy loading of the long side groups causes their hydrogen bonding that prevent from the flexible adapting of the shape for the guest strong binding. In only five of the investigated structures the spatial arrangement of the functional groups of DFH allows for the intramolecular hydrogen bond formation of the N–H...O type, namely CS5(NH${}_{2}$)${}_{3}$:DFH**10**, CS5(NH${}_{2}$)${}_{3}$:DFH**20**, CS5(NH${}_{2}$)${}_{3}$:DFH**19**, CS5(NH${}_{2}$)${}_{3}$:DFH**2** and CS5(NH${}_{2}$)${}_{3}$:DFH**1** (see Figure 16 or Supporting Information).

The energy scale found for the CS5(NH${}_{2}$)${}_{3}$:DFH complexes is similar as in the above discussed modified chitosan interactions and ranges from −48.76 kcal/mol for the most stable CS5(NH${}_{2}$)${}_{3}$:DFH**10** to −14.80 kcal/mol for CS5(NH${}_{2}$)${}_{3}$:DFH**14** with the exception of additionally stabilized CS5(NH${}_{2}$)${}_{3}$:DFH**13-PT** exhibiting the carboxyl to amino group proton transfer between the subsystems (interaction energy −103.76 kcal/mol). In the least stable structure, CS5(NH${}_{2}$)${}_{3}$:DFH**14**, the intermolecular interactions are acting for the longer distance than in conventional hydrogen bonds. Moreover, diclofenac is placed on the edge of the polymeric system rather than on its sheet, thus minimizing the possibility of any other weak interactions.

### 2.4. Chitosan Dimers

**Figure 17:**
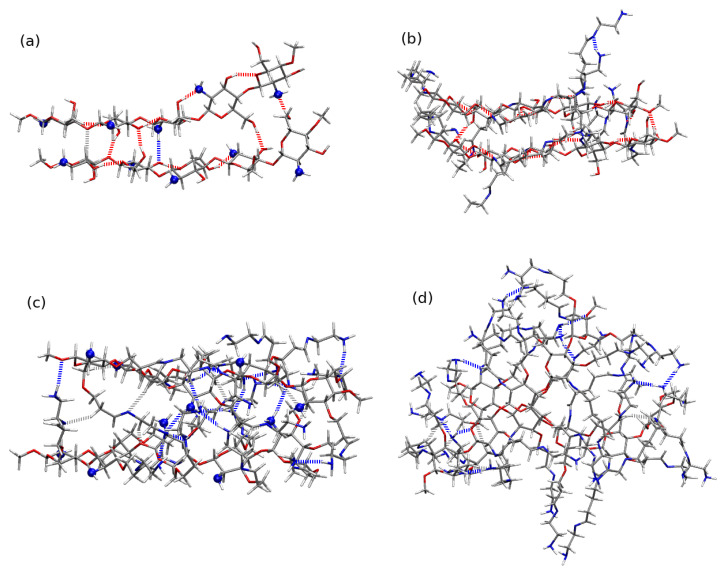
Most attractive B97-D3/6-31G(d,p) optimized chitosan dimers for (**a**) ${\left[CS5\right]}_{2}$, (**b**) ${\left[CS5(NH_{2})\right]}_{2}$, (**c**) ${\left[CS5{\left(NH_{2}\right)}_{2}\right]}_{2}$, (**d**) ${\left[CS5{\left(NH_{2}\right)}_{3}\right]}_{2}$. The blue balls represent nitrogens in the unmodified amino groups close to the polymer backbone and dashed lines are the hydrogen bonds.

In order to investigate the competitiveness of the mutual interaction between two chitosan chains and chitosan with diclofenac, the exemplary calculations of the chitosan dimers were performed for the long five-membered chains, both pristine and modified. All the optimized structures corresponding to the minima on the potential energy surface are presented in Supporting Information and here only the most stable dimers are shown in Figure 17. In the case of the pristine chitosan chain one can see that the amino groups close to the polymer backbone take active part in the aggregation process, creating the hydrogen bonds between the monomers. The case of ${\left[CS5{\left(NH_{2}\right)}_{2}\right]}_{2}$ is not that obvious, however the unmodified amino groups provide the hydrogen bond donor or/and acceptor both for intermolecular and intramolecular stabilizing interactions. For the remaining dimers, built of the chains with modified amino groups close to the backbone, one can notice that the mutual interaction between chains is mostly governed by the weak electrostatic interactions, and most hydrogen bonds occur intramolecularly in the monomers.

Supermolecular energy for all the investigated dimers is presented in Table 4. Comparison of these data with the chitosan–diclofenac interaction energies presented in Table 3 allows to clearly state that due to the smaller number of H-bond and other electrostatic contacts in the interaction of chitosan with diclofenac in comparison to the chitosan dimer, the interaction energy ranges are significantly smaller in drug–biopolymer interaction than in biopolymer dimers. Thus, one can expect that in the case of the diclofenac entering the real biopolymer material, its interaction with chitosan will not be strong enough to move the chains apart and penetrate inside. Rather, diclofenac will be prone to the interactions with chitosan external functional groups on the surface of the material.

**Figure 18:**
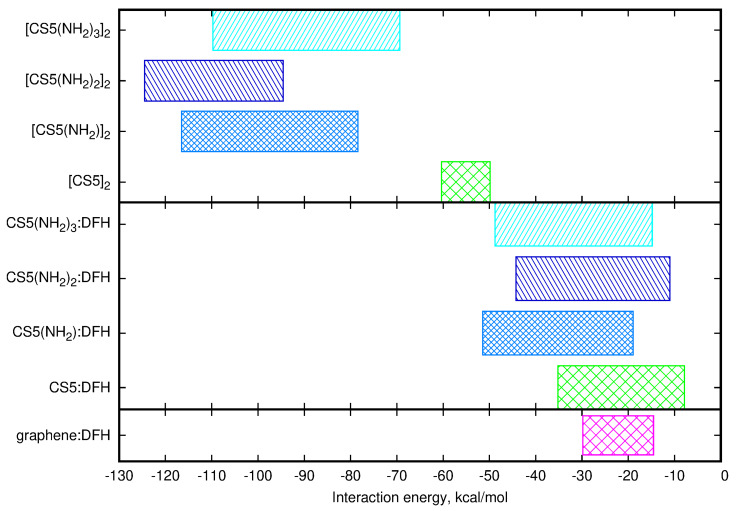
Supermolecular interaction energy ranges for chitosan dimers (upper panel), diclofenac:chitosan (middle panel) and diclofenac:graphene complexes [61] (lowest panel).

Indeed, a closer look to the optimized dimer structures, confirms the above statements and remains in agreement with our previous molecular dynamics study [27]. The unmodified chitosan dimers [CS5]${}_{2}$, containing one amino group and two hydroxyl groups per unit, are likely to adapt the parallel structure with some geometrical modifications arising from the tendency to the helical arrangements. The differences between the investigated dimers do not exceed 20% of highest obtained interaction energy for these systems. Among the three substituted chitosan derivatives, most stable dimers are formed by the doubly modified [CS5(NH${}_{2}$)${}_{2}$]${}_{2}$. This may be an artefact of the limited number of configurations taken into account, however, considering the growing amount of potential H-bonding donors and acceptors with the increasing number of long substituents in a chain, the following order of the intermolecular interaction energy between the two chitosan chains: ${\left[CS5{\left(NH_{2}\right)}_{2}\right]}_{2}>{\left[CS5{\left(NH_{2}\right)}_{3}\right]}_{2}>{\left[CS5(NH_{2})\right]}_{2}>{\left[CS5\right]}_{2}$ seems to be not an artificial outcome of the imperfect model. This trend remains in agreement with our previous findings that the large number of the strong hydrogen bonds of the N–H...O type close to the polymer backbone can result in more synchronized chain structuring and therefore in dense polymer aggregates. Moreover, this tendency for chitosan dimer interaction energy is opposite to the one noticed for the chitosan chain–diclofenac interaction, for which intermolecular interaction energy changes in the following order: ${\left[CS5(NH_{2})\right]}_{2}>{\left[CS5{\left(NH_{2}\right)}_{3}\right]}_{2}>{\left[CS5{\left(NH_{2}\right)}_{2}\right]}_{2}>{\left[CS5\right]}_{2}$. All of these trends are clearly visible in Figure 18, where the mutual attraction in ${\left[CS5{\left(NH_{2}\right)}_{3}\right]}_{2}$, depicted as dark blue box in the upper panel, is most shifted into the stronger interaction side. Additionally, due to the smaller size of the DFH molecular than the second chitosan chain in a dimer, the interactions of DFH:chitosan are significantly lower than in a homogeneous biopolymer dimers.

For completeness, the lowest panel of Figure 18 presents the supermolecular B97-D3/jun-pVDZ energy range obtained in our previous study [61]. This range regards different structural modifications of an analyzed carbon material: from carboxylated graphene with the interaction energy toward DFH equal to −14.55 kcal/mol to a carboxylated material with double vacancies, significantly deformed from planarity, exhibiting −29.81 kcal/mol attraction toward the drug. One can easily estimate that with respect to the single limited-size graphene surface, chitosan can provide a competitive alternative in the drug adsorption processes due to the higher interaction energy upon an adequate modification. It should also be noticed that the nature of the diclofenac interaction is different for both of these types of adsorbents: on graphene dispersion forces predominate and determine the sandwich-like geometry of the π−π complexes, while for chitosan-type materials electrostatic component is crucial and hydrogen bonds create a net of attractive contacts between material and the drug.

## 3. Discussion

The present study constitutes the quantitative gas phase computational contribution devoted to the feasibility of application of pristine and modified chitosan materials previously obtained in our group to the adsorption of non-steroidal anti-inflammatory drugs from wastewater. Namely, the broad study of the interaction of the chitosan models with diclofenac is presented. Supermolecular calculations allow to determine the most important factors in the mutual chitosan unit:diclofenac contacts, i.e., the net of the hydrogen bonds and the potential presence of the cation in the middle of the interaction center. The three different variants of CS models: single-unit, five-units and chemically modified allow to obtain qualitatively similar results, being moreover consistent almost independently on the applied approach. Therefore, one can assume that even the smallest model can provide valuable data for the investigation of structural and energetic aspect of chitosan interaction with small molecules. One need to remember however, that such a short chitosan model would not be able to reproduce the possible pore formation or strength of aggregation phenomena observed for the longer biopolymeric chains [27]. Up-to-date studies show undoubtedly that the main problem of any practical diclofenac application, namely its poor solubility, can be overcome with the usage of its sodium or potassium salts. These also can improve its physisorption on the materials applied in various branches of technology, such as carbon nanomaterials containing any functional groups or biopolymers.

The present results clearly show that such a complex phenomenon as the different affinity of (modified) chitosan chains with respect to diclofenac in any form cannot be fully explained by such a reduced model. In the real system, first of all only three of each four rings are deacetylated and the fourth one remains in the unchanged chitin form. This influences the solubility of the system, since chitin is barely soluble in investigated solvents, and can also affect the possibility of material modification on chitin rings. The only obvious difference between CS2 and the remaining modified materials is the presence of the single amino group close to each of its rings. This may influence the hydrogen bond network in the system and cause stronger binding between the material chains. The above remarks are confirmed by the interaction energies obtained for the chitosan chain dimers. Supermolecular approach applied for these large molecular complexes point out undoubtly that the system with two modified hydroxyl groups and one pristine amino group close to the polymer backbone exhibit the strongest attraction in dimer. This interaction is also two to three times stronger than for the respective chitosan chain with diclofenac, depending on the particular biopolymer modification. Therefore one can assume that diclofenac penetration into the chitosan aggregates will be hindered by the substantial mutual biopolymer chain interactions and the chitosan materials of the investigated form will not provide the optimal NSAIDs adsorbents. Thus, other biopolymer modifications are highly desirable that decrease its self-aggregation, increase its pore (or in general surface) volume or exploit different mechanism of drug:material interaction.

On the other hand, comparison of the results of the present study to our previous study for diclofenac physisorption on graphene allows to assume that chitosan may be still a feasible adsorbent. SCS-SAPT0 interaction energies for graphene–diclofenac attraction are in the range of −16 to −11 kcal/mol, depending on the surface modification, and strong dispersion character is noticed for all of the analyzed structures [61]. Contrarily, in chitosan–diclofenac complexes, for pristine biopolymer chains interactions of the order of −30 kcal/mol are available and the supermolecular approach shows that they can increase to −40 or even −50 kcal/mol upon chitosan grafting with long alkyl substituents. Thus, the current data together with the previous literature reports [14] support the further study of the chitosan modification for the improvement of its adsorption capacity with respect to non-steroidal anti-inflammatory drugs.

It needs to be underlined again that the current study presents only gas phase quantum mechanical results, which can strongly affect the obtained data both geometrically and energetically. Our preliminary tests however proved that the application of polarizable continuum model leads to the qualitatively similar results at least for the smallest of the investigated systems. The advanced molecular dynamics study involving the long chitosan chains interacting with diclofenac in solvent is in progress and will be published soon.

## 4. Materials and Methods

Three models of chitosan have been taken into account: single pristine chitosan unit (CS1), a pristine chain constructed of five units (CS5) and a five units chain substituted with the long amino side chains in positions of –NH${}_{2}$ and –OH chitosan groups. Substituted models are constructed according to Ref. [18] and can be considered in three groups: single amino group substituted by the long imine-amine side chain (CS5(NH${}_{2}$)), two hydroxyl groups substituted (CS5(NH${}_{2}$)${}_{2}$) and all –OH and –NH${}_{2}$ in an original CS5 substituted (CS5(NH${}_{2}$)${}_{3}$). All the analyzed chitosan models are presented in Figure 9.

For the smallest model the thorough investigation has been undertaken that covers the influence of the form of diclofenac on the mutual material:drug interaction. Namely, diclofenac in its acidic neutral form (DFH), diclofenac anion (DF${}^{-}$) and diclofenac sodium (DFNa) are examined. Ten initial structures analogous for all forms of a drug have been analyzed for each complex in order to avoid of the encountering of an artificial local minimum. In the case of chitosan interactions with anion, several initial complexes has led to the structures resulting from the proton transfer from chitosan moiety to an anion and these were also commented upon in further considerations.

Due to the large size of the destination systems, namely long chain substituted chitosan derivatives, the main part of geometry optimization calculations has been performed in B97-D3/6-31G(d,p) approach for all complexes. Additionally, for single pristine unsubstituted chitosan unit interacting with diclofenac anion in order to verify the correct performance of the small basis sets, the tests in 6-311++G(d,p) and 6-311++(2df,2pd) basis sets have been carried out, taking into account the diffused nature of the anionic electron distribution. The obtained results do not differ much from the basic structures and thus were omitted in further part of this study. The character of the obtained stationary points is confirmed by the harmonic vibrational analysis.

Counterpoise-corrected interaction energy in a chitosan:drug complexes has been estimated with the supermolecular approach according to the following expression:

(1) $$\begin{array}{c} ∆E_{SM}^{CP}=E_{complex}\left(complex\right)-\left[E_{chitosan}\left(complex\right)+E_{drug}\left(complex\right)\right], \end{array}$$

where $E_{X}\left(complex\right)$ denotes the energy of system X (X being complex, chitosan or the drug) in the basis set of the whole complex [62,63,64,65]. B97-D3 and ωB97X-D DFT functionals are applied for their well-known good behavior in the description of the weak intermolecular interactions [66]. Moreover, the perturbation theory has been also tested. For its overbinding in estimation of molecular attraction by conventional MP2 approach [67,68,69,70], also MP2-coupled (MP2C) version was employed [71,72,73]. This method developed by Hesselmann directly corrects the supermolecular MP2 interaction energy with the additional intramonomer contribution to the dispersion energy term calculated with TD-DFT.

For the better understanding of the physical nature of chitosan:diclofenac interaction, the interaction energy partitioning has been performed with the SAPT0 approach [55,56,57,58,59] for CS1 system interactions with all the analyzed forms of the drug and for the interaction of CS5 with DFH molecule.

The electrostatic-to-dispersion ratio (ELST/DISP) is applied as considered by Rezač et al. [60]. In order to classify the complexes into one of three classes: dispersion-dominated, electrostatic-dominated or mixed. The first class of complexes is characterized by the value of ELST/DISP smaller than 0.59 and the second—larger than $\frac{1}{0.59}=1.7$.

For single chitosan ring interacting with diclofenac moieties, NCI analysis of non-covalent interactions based on electron density and its reduced gradient has been performed and its effects are visualized with NCIPLOT 3.0 [74,75].

A set of different Pople and Dunning basis sets of double-zeta and triple-zeta quality (namely, 6-311++G(d,p), cc-pVDZ, aug-cc-pVDZ, aug-cc-pVTZ) was applied for supermolecular interaction energy estimation as well as for SAPT0 calculations for smaller investigated complexes. Large substituted chitosan chains were treated with 6-311++G(d,p) basis set only.

Additionally, in order to investigate the competitiveness between the diclofenac:chitosan and chitosan:chitosan interactions, also the representative chitosan dimers were included into the present study. The full geometry optimization together with the harmonic vibrational analysis has been performed within the B97X-D3/6-31G(d,p) approach. The supermolecular interaction energies were also corrected by the counterpoise procedure.

All the geometry optimizations and DFT interaction energies were obtained with Gaussian16 [76], MP2C calculations were performed with Molpro2018.1 [77,78] and SAPT0 estimations were carried out with PSI4 package [79,80].

## Figures and Tables

**Table 1:** B97-D3/6-311++G(d,p), ωB97X-D/6-311++G(d,p), MP2/6-311++G(d,p), MP2/aug-cc-pVDZ counterpoise corrected interaction energies [kcal/mol] and MP2C/aug-cc-pVDZ and SAPT0/cc-pVDZ and SAPT0/aug-cc-pVDZ total interaction energy single chitosan unit CS1 with DFNa, DF${}^{-}$ and DFH (bold: most attractive B97-D3 interaction; italic: weakest B97-D3 interaction). P++(d,p) denotes Pople 6-311++G(d,p) basis set, DZ is Dunning cc-pVDZ basis set and aDZ is Dunning DZ augmented with a set of diffuse functions (aug-cc-pVDZ). For the system structures see Figure 1, Figure 3 and Figure 4 and Supplementary Materials.

Approach	B97-D3	ωB97X-D	MP2	MP2	MP2C	SAPT0	SAPT0
Basis Set	P++(d,p)	P++(d,p)	P++(d,p)	aDZ	aDZ	DZ	aDZ
CS1:DFNa							
**1**	−36.10	−38.68	−31.08	−33.34		−34.66	−39.20
2	−25.54	−27.82	−21.40	−23.66		−25.24	−29.62
3	−23.94	−25.72	−17.51	−19.97		−19.72	−24.33
**4**	−38.46	−41.20	−32.89	−35.76		−37.12	−42.32
5	−33.23	−36.00	−27.63	−30.74		−30.46	−36.51
**6**	−34.84	−36.72	−29.59	−31.64		−33.12	−37.19
7	−24.45	−25.96	−19.64	−20.89		−23.13	−25.90
7a	−32.29	−34.92	−26.38	−29.21		−31.76	−36.76
8	−21.79	−22.61	−17.05	−18.93		−19.60	−23.10
**9**	−34.19	−35.50	−28.65	−30.40		−31.93	−35.66
*10*	−9.74	−10.32	−7.87	−10.31		−6.98	−11.99
CS1:DF${}^{-}$							
2	−24.11	−25.60	−21.56			−23.32	−30.24
**3**	−28.99	−32.46	−27.28			−30.01	−36.24
**4**	−27.56	−30.00	−24.86			−27.46	−33.64
6	−25.72	−27.79	−23.87			−26.54	−30.92
**7a**	−27.92	−30.91	−26.28			−30.11	−34.40
*8*	−21.47	−22.60	−19.13			−19.80	−25.60
CS1:DFH							
2	−19.04	−20.71	−15.35	−18.80	−16.99	−17.20	−23.63
*3*	−5.99	−6.73	−4.50	−5.76	−5.26	−4.47	−6.93
**4**	−20.06	−22.02	−16.73	−19.77	−17.75	−18.32	−24.24
5	−8.78	−9.31	−10.80	−9.38	−7.74	−6.35	−10.91
**6**	−26.48	−27.52	−22.21	−25.59	−23.58	−23.99	−30.94
7	−11.74	−13.34	−9.00	−11.84	−9.49	−8.24	−13.85
8	−11.33	−13.23	−9.36	−12.18	−9.95	−9.13	−15.09
**9**	−20.77	−22.54	−16.86	−19.82	−17.86	−18.33	−24.32

**Table 2:** SAPT0/aug-cc-pVDZ interaction energy components for interaction of single chitosan unit CS1 with DFNa, DF${}^{-}$ and DFH (boldfaced most attractive SAPT0 interaction, boxes for ELST/DISP ratio smaller than 0.59, denoting dispersion-dominated systems and underlined energies for ELST/DISP ratio exceeding 1.7, denoting electrostatic-dominated systems according to Ref. [60]; for the system structures see Figure 1, Figure 3 and Figure 4 and Supplementary Materials).

System	SAPT0	SCS-SAPT0	ELST	EXCH	IND	DISP	SCS-DISP	ELST/DISP	ELST/SCS-DISP
CS1:DFNa									
**1**	**−39.20**	−34.33	−35.16	26.85	−8.74	−22.16	−17.29	1.59	2.03
2	−29.62	−25.23	−34.15	33.82	−9.13	−20.17	−15.78	1.69	2.16
3	−24.33	−20.22	−21.81	25.23	−8.93	−18.83	−14.71	1.16	1.48
**4**	**−42.32**	−37.36	−49.26	41.77	−12.01	−22.82	−17.86	2.16	2.76
5	−36.51	−31.27	−35.93	32.66	−9.32	−23.91	−18.67	1.50	1.93
**6**	**−37.19**	−33.01	−37.73	28.35	−8.67	−19.14	−14.95	1.97	2.52
7	−25.90	−22.79	−25.57	20.08	−6.27	−14.13	−11.02	1.81	2.32
**7a**	**−36.76**	−31.74	−48.42	51.43	−16.52	−23.25	−18.23	2.08	2.66
8	−23.10	−19.51	−22.40	20.67	−5.13	−16.24	−12.65	1.38	1.77
**9**	**−35.66**	−31.79	−33.83	25.08	−9.18	−17.73	−13.86	1.91	2.44
*10*	*−11.99*	−8.11	−8.91	17.32	−2.84	−17.56	−13.69	0.51	0.65
CS1:DF${}^{-}$									
2	−30.25	−25.39	−29.07	37.47	−16.39	−22.26	−17.41	1.31	1.67
**3**	**−36.24**	−31.42	−39.32	40.69	−15.34	−22.27	−17.45	1.77	2.25
**4**	**−33.64**	−28.41	−29.41	31.60	−11.88	−23.96	−18.73	1.23	1.57
6	−30.92	−27.43	−29.17	26.50	−12.24	−16.01	−12.52	1.82	2.33
**7a**	**−34.40**	−30.71	−34.54	32.32	−15.04	−17.13	−13.44	2.02	2.57
*8*	*−25.60*	−21.07	−23.67	28.47	−9.77	−20.63	−16.10	1.15	1.47
9	−30.91	−27.42	−29.17	26.50	−12.23	−16.00	−12.52	1.82	2.33
CS1:DFH									
**2**	**−23.63**	−18.49	−30.92	42.40	−11.47	−23.63	−18.49	1.31	1.67
*3*	*−6.93*	−4.67	−6.47	11.59	−1.80	−10.25	−7.99	0.63	0.81
**4**	**−24.24**	−19.31	−23.45	30.65	−9.00	−22.44	−17.51	1.05	1.34
5	−10.91	−7.45	−6.84	13.46	−1.96	−15.58	−12.12	0.44	0.56
**6**	**−30.94**	−25.46	−37.00	51.19	−19.74	−25.40	−19.91	1.46	1.86
7	−13.85	−9.02	−7.41	18.23	−2.83	−21.85	−17.02	0.34	0.44
8	−15.09	−10.45	−12.85	22.01	−3.13	−21.12	−16.47	0.61	0.78
**9**	**−24.32**	−19.28	−26.47	36.91	−11.67	−23.09	−18.05	1.15	1.47

**Table 3:** B97-D3/6-311++G(d,p) supermolecular counterpoise-corrected interaction energy [kcal/mol] in CS5(NH${}_{2}$)${}_{x}$:DFH complexes for x=1,2,3. PT in superscript denoted the structure with intermolecular proton transfer.

System	CS5(NH${}_{2}$):DFH	CS5(NH${}_{2}$)${}_{2}$:DFH	CS5(NH${}_{2}$)${}_{3}$:DFH
1	−38.93	−35.66	−22.95
2	−18.93	−31.55	−34.41
3	−41.04	−19.49	−27.27
4	−29.20	−14.79	−22.31
5	−51.45	−10.91	−34.86
6	−30.42	−34.08	−32.82
7	−40.08	−26.81	−18.93
8	−35.45	−40.81	−38.93
9	−32.36	−37.22	−37.04
10	−37.09	−40.86	−48.76
11	−35.79	−28.40	−45.51
12	−36.24	−19.64	−46.27
13	−21.03	−36.81	−103.76${}^{PT}$
14	−31.09	−44.26	−14.80
15	−29.30	−30.52	−28.83
16	−35.72	−29.02	−25.21
17	−35.42	−30.82	−38.17
18	−31.42	−22.55	−46.66
19	−36.18	−31.67	−39.11
20	−27.00	−31.48	−47.92

**Table 4:** Supermolecular interaction energies [kcal/mol] for chitosan dimers calculated with B97-D3/6-31G(d,p) approach in vacuum. For comparison, the ranges of diclofenac–material interaction energies from Table 3 are given in the last column.

Material	Dimer 1	Dimer 2	Dimer 3	Dimer 4	Dimer 5	Diclofenac
[CS5]${}_{2}$	−60.32	−59.64	−51.61	−61.25	−49.84	−7.86 to −35.19
[CS5(NH${}_{2}$)]${}_{2}$	−116.50	−102.48	−81.53	−78.43	−107.35	−18.93 to −51.45
[CS5(NH${}_{2}$)${}_{2}$]${}_{2}$	−124.50	−94.55	−103.94			−10.91 to −44.26
[CS5(NH${}_{2}$)${}_{3}$]${}_{2}$	−109.76	−69.34	−99.19			−14.80 to −48.76

## Supplementary Materials

The following are available online.

## Abbreviations

The following abbreviations are used in this manuscript:

COX	cyclooxygenase
NSAID	Non-Steroidal Anti-Inflammatory Drugs
CS	Chitosan
DFH	Diclofenac
DFT	Density Functional Theory
SAPT	Symmetry-Adapted Perturbation Theory
CPK	Corey–Pauling–Koltun Representation
NCI	Non-Covalent Interactions
NCIPLOT	Non-Covalent Interaction PLOTting program
