# Supplementary material for: Gas Phase Computational Study of Diclofenac Adsorption on Chitosan Materials
Source: Molecules. 2020 May 30;25(11):2549. doi: 10.3390/molecules25112549 (PMC7321203; doi:10.3390/molecules25112549)
Supplement: Supplementary file 1 [file molecules-25-02549-s001.pdf]

# Supporting Information:

# Feasibility of chitosan application for wastewater purification from diclofenac


Anna Kaczmarek-Kędziera*

*Faculty of Chemistry, Nicolaus Copernicus University in Toruń, Gagarina 7, 87–100 Toruń, Poland*

E-mail: teoadk@chem.umk.pl


Table S1: Basis set dependence of interaction energy for CS1:DFNa complexes in B97-D3 functional. P(d,p) denotes 6-31G(d,p) Pople basis set, P++(d,p) stands for Pople 6-311++G(d,p) basis set, P++(2df,2pd) is 6-311++G(2df,2pd) basis set. #bf is a number of basis functions for the corresponding basis set.

| Basis set | P(d,p) | P++(d,p) | P++(2df,2pd) |
|---|---|---|---|
| #bf | 657 | 961 | 1585 |
| B97-D3 | | | |
| **1** | -37.14 | -36.10 | -35.43 |
| 2 | -26.58 | -25.54 | -25.02 |
| 3 | -24.84 | -23.94 | -23.84 |
| **4** | -40.43 | -38.46 | -38.12 |
| 5 | -34.78 | -33.23 | -32.88 |
| **6** | -36.44 | -34.84 | -34.28 |
| 7 | -24.64 | -24.45 | -23.86 |
| 7a | -33.70 | -32.29 | -31.79 |
| 8 | -22.76 | -21.79 | -21.64 |
| **9** | -35.68 | -34.19 | -33.57 |
| *10* | -9.51 | -9.74 | -9.82 |

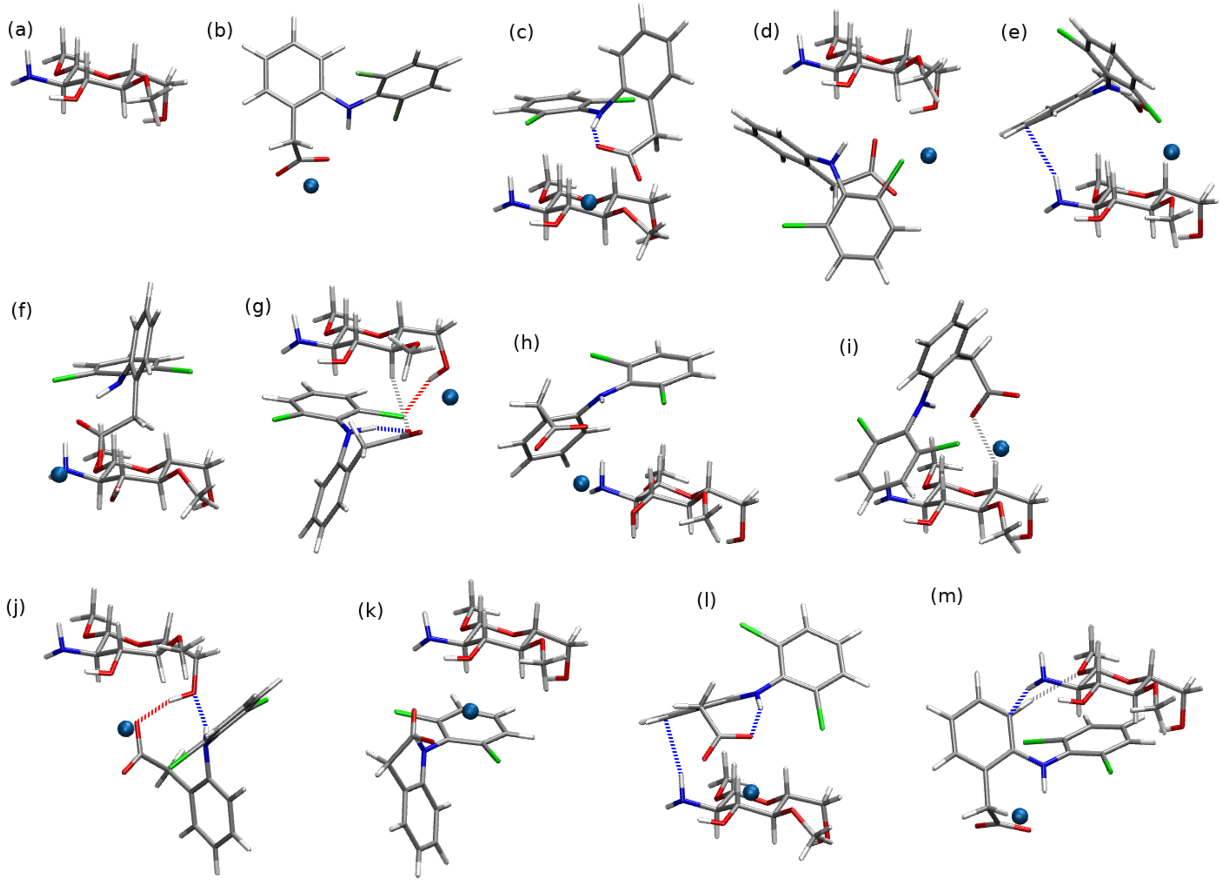


Figure S1: Optimized geometries for diclofenac sodium DFNa interacting with pristine chitosan unit CS1: (a) optimized chitosan unit CS1 – lowest energy conformation, (b) optimized diclofenac sodium molecule DFNa and CS1:DFNa complexes: (c) **1**, (d) **2**, (e) **3**, (f) **4**, (g) **5**, (h) **6**, (i) **7**, (j) **7a**, (k) **8**, (l) **9**, (m) **10** (carbon atoms – grey, hydrogen – white, nitrogen – blue, oxygen – red, chlorine – green; sodium cation presented as blue balls and H-bonds – dashed).

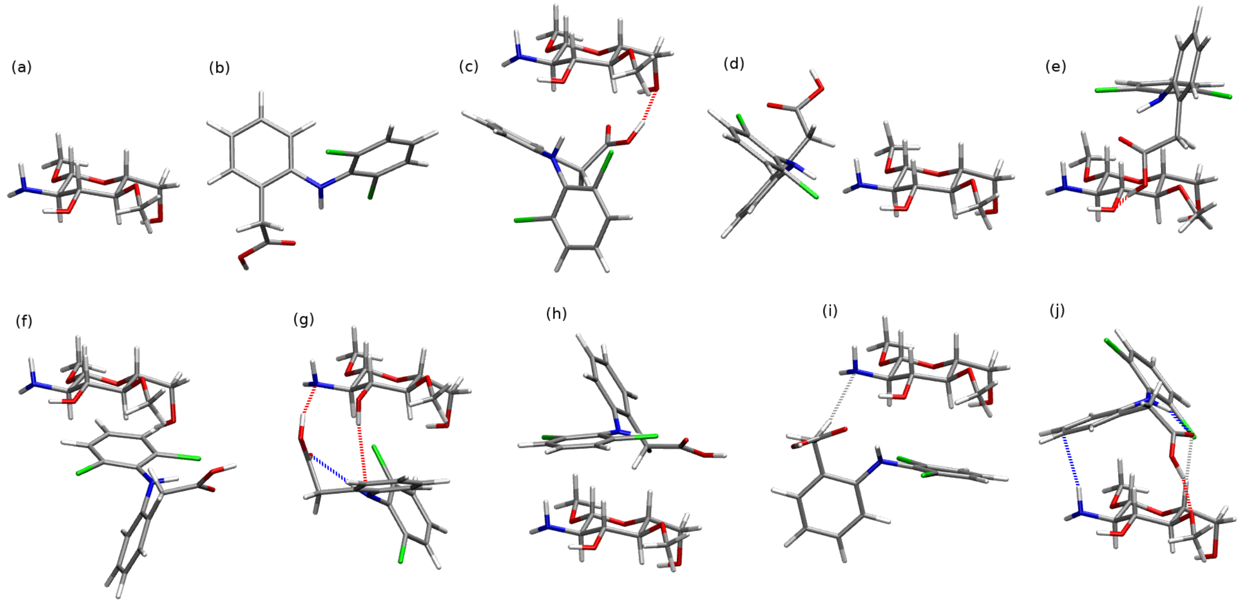


Figure S2: Optimized geometries for diclofenac (acidic form) DFH interacting with pristine chitosan unit CS1: (a) optimized chitosan unit CS1 – lowest energy conformation, (b) optimized diclofenac molecule DFH and CS1:DFH complexes: (c) **2**, (d) **3**, (e) **4**, (f) **5**, (g) **6**, (h) **7**, (i) **8**, (j) **9** (carbon atoms – grey, hydrogen – white, nitrogen – blue, oxygen – red, chlorine – green; sodium cation presented as blue balls and H-bonds – dashed).

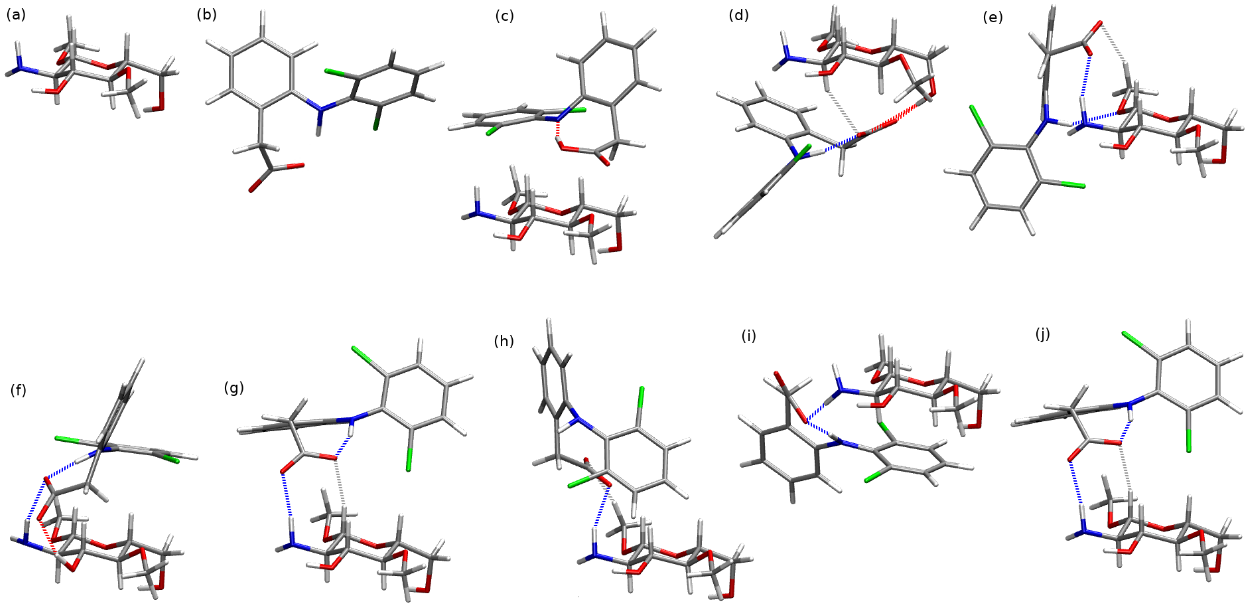


Figure S3: Optimized geometries for diclofenac anion $DF^-$ interacting with pristine chitosan unit CS1: (a) optimized chitosan unit CS1 – lowest energy conformation, (b) optimized diclofenac anion $DF^-$ and CS1:$DF^-$ complexes: (c) **1-PT**, (d) **2**, (e) **3**, (f) **4**, (g) **6**, (h) **7a**, (i) **8**, (j) **9** (carbon atoms – grey, hydrogen – white, nitrogen – blue, oxygen – red, chlorine – green; sodium cation presented as blue balls and H-bonds – dashed).

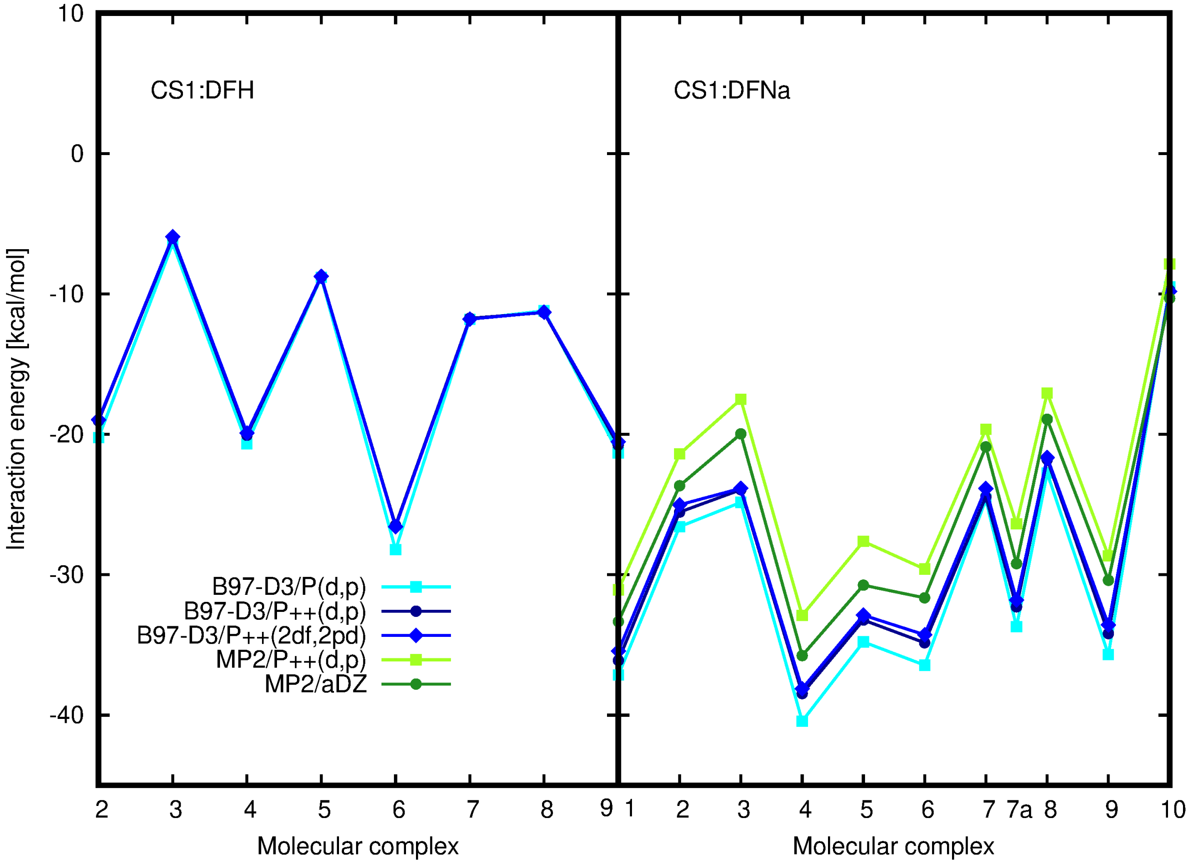


Figure S4: Basis set dependence of B97-D3 and MP2 supermolecular interaction energy for CS1:DFH and CS1:DFNa complexes

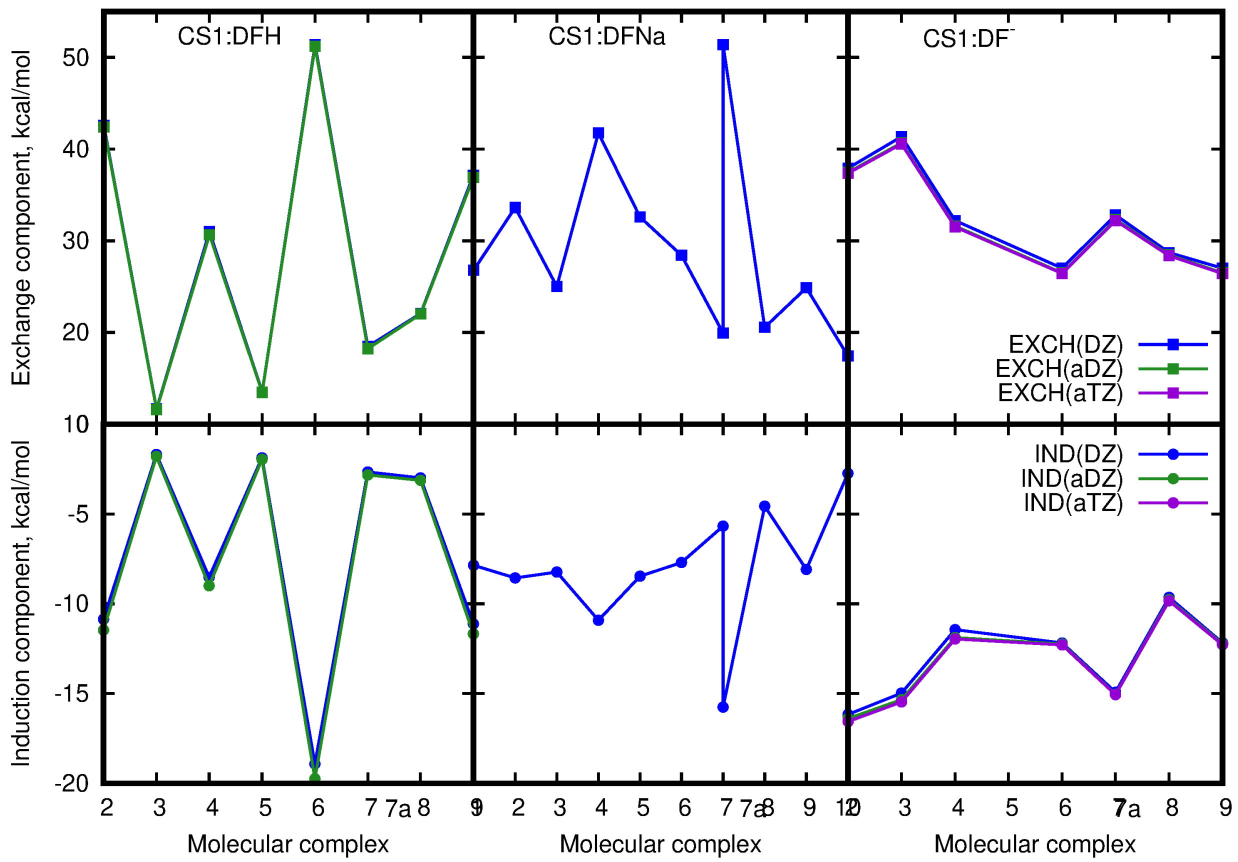


Figure S5: Induction and exchange SAPT0 interaction energy components for CS1:drug complexes

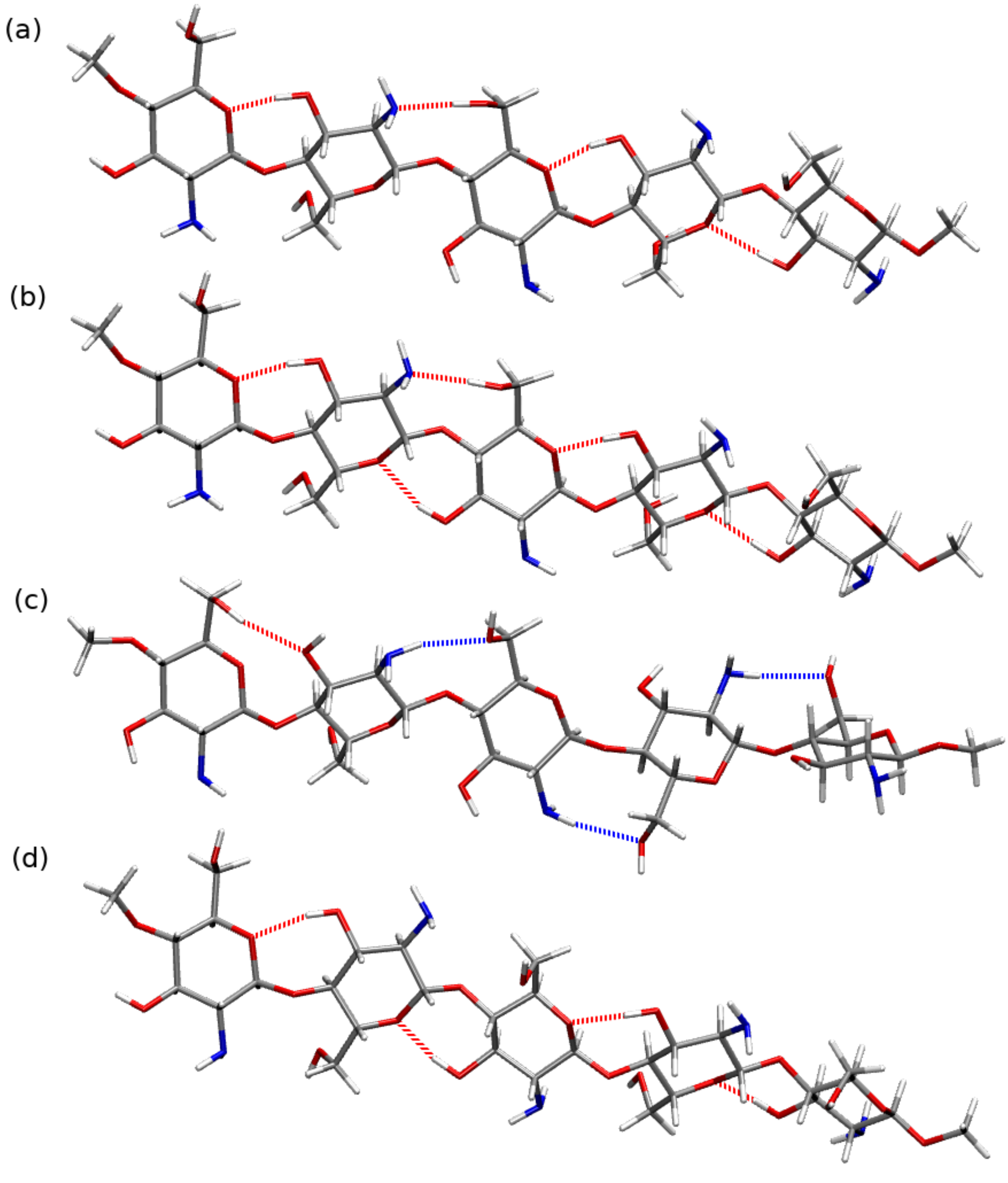


Figure S6: Optimized geometries for exemplary chitosan five-unit chains from different initial points. Relative B3LYP/6-31G(d,p) energy: (a) 0.00 kcal/mol, (b) 1.01 kcal/mol, (c) 8.96 kcal/mol and (d) 9.19 kcal/mol.

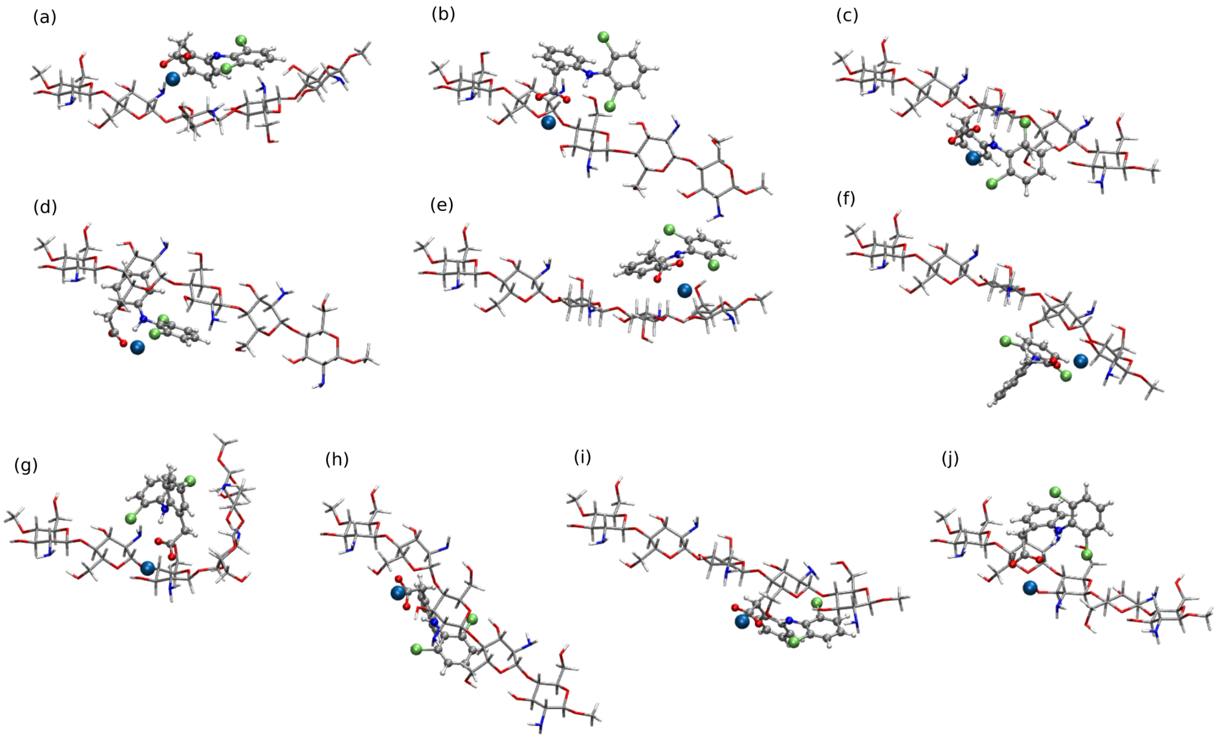


Figure S7: Optimized geometries for diclofenac sodium DFNa interacting with pristine chitosan unit CS5. CS5:DFNa complexes: (a) **1**, (b) **2**, (c) **3**, (d) **4**, (e) **5**, (f) **6**, (g) **7**, (h) **8**, (i) **9**, (j) **10** (carbon atoms – grey, hydrogen – white, nitrogen – blue, oxygen – red, chlorine – green; sodium cation presented as blue balls and H-bonds – dashed).

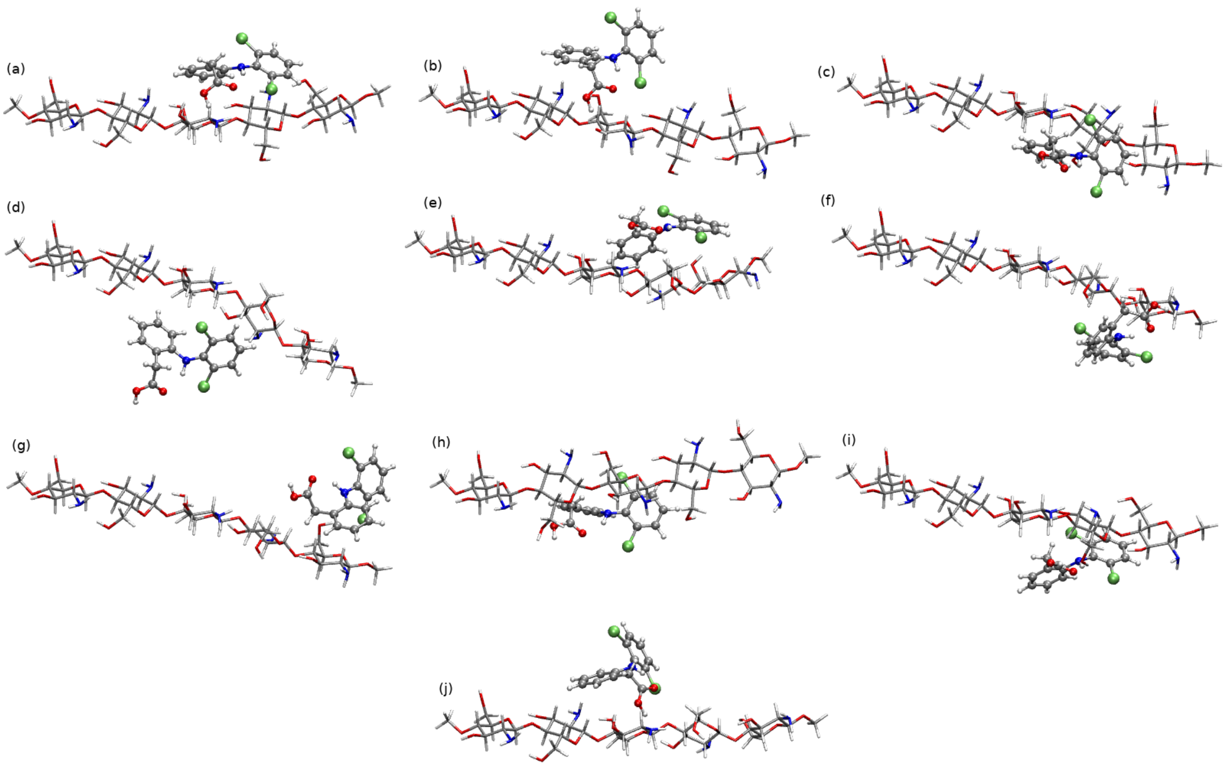


Figure S8: Optimized geometries for diclofenac DFH interacting with pristine chitosan unit CS5. CS5:DFH complexes: (a) **1**, (b) **2**, (c) **3**, (d) **4**, (e) **5**, (f) **6**, (g) **7**, (h) **8**, (i) **9**, (j) **10** (carbon atoms – grey, hydrogen – white, nitrogen – blue, oxygen – red, chlorine – green; sodium cation presented as blue balls and H-bonds – dashed).

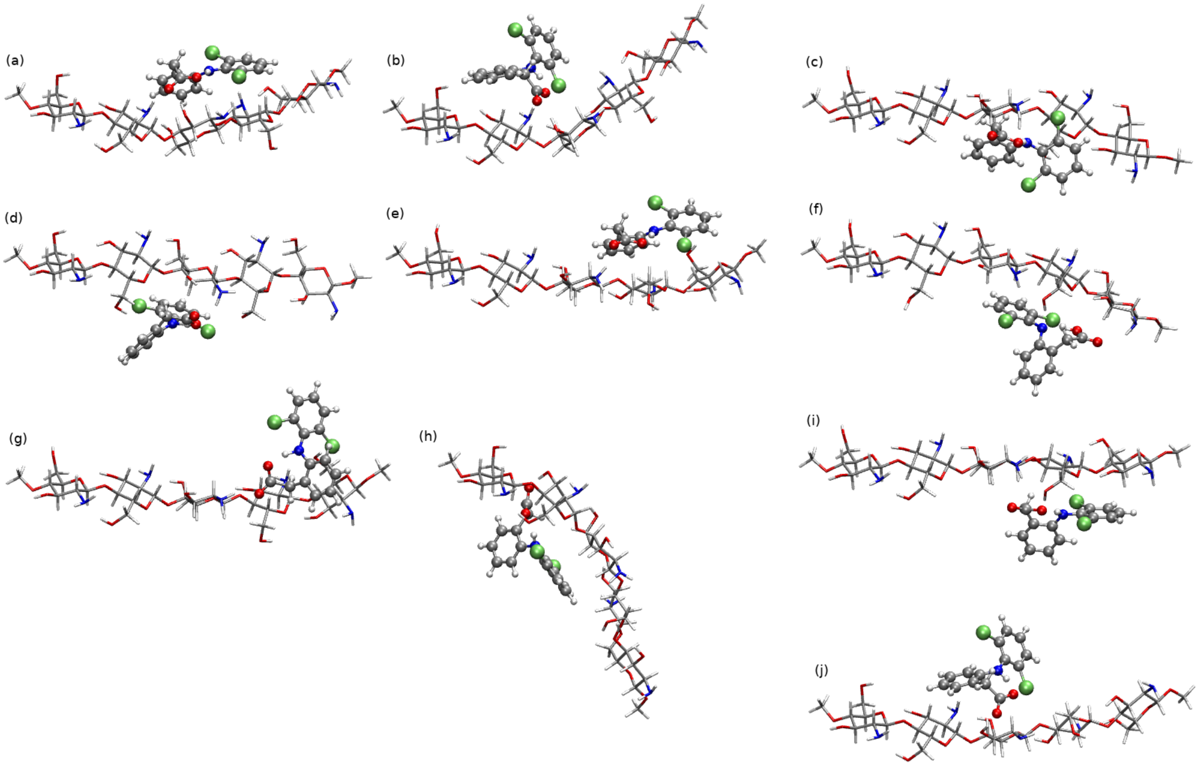


Figure S9: Optimized geometries for diclofenac anion interacting with pristine chitosan unit CS5. CS5:DF$^{-}$ complexes: (a) **1**, (b) **2**, (c) **3**, (d) **4**, (e) **5**, (f) **6**, (g) **7**, (h) **8**, (i) **9**, (j) **10** (carbon atoms – grey, hydrogen – white, nitrogen – blue, oxygen – red, chlorine – green; sodium cation presented as blue balls and H-bonds – dashed).

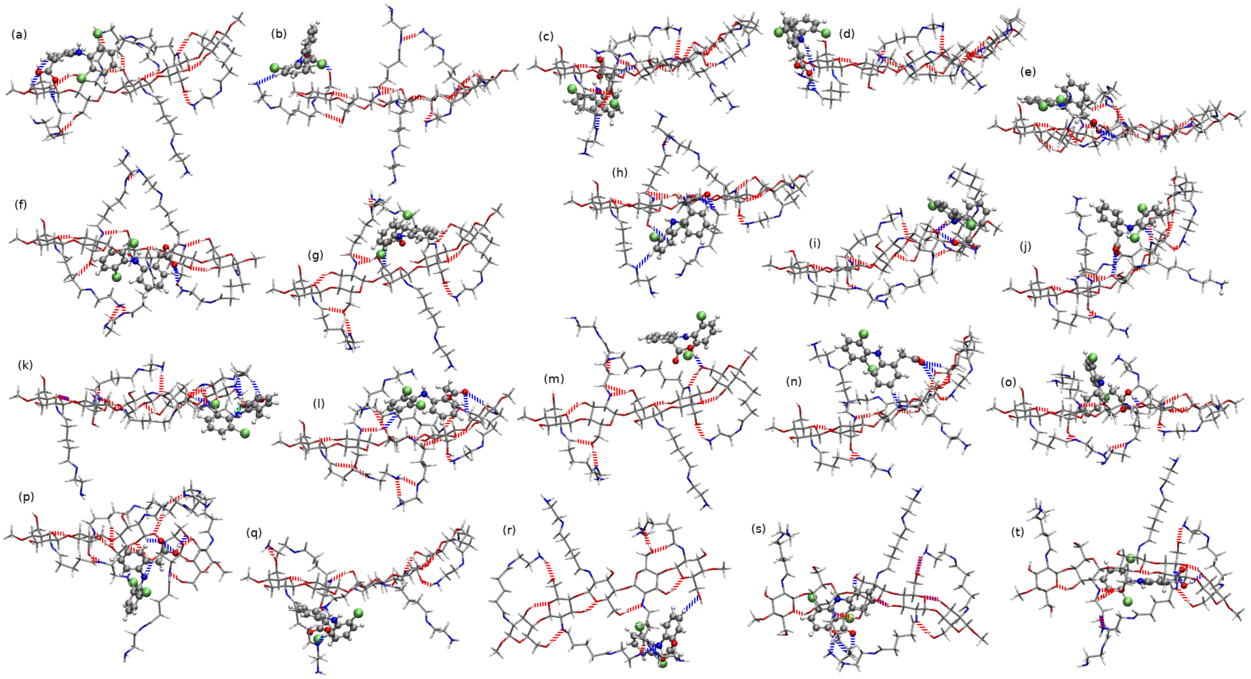


Figure S10: Optimized geometries for diclofenac acid DFH interacting with substituted chitosan unit CS5($NH_2$). (carbon atoms – grey, hydrogen – white, nitrogen – blue, oxygen – red, chlorine – green; sodium cation presented as blue balls and H-bonds – dashed).

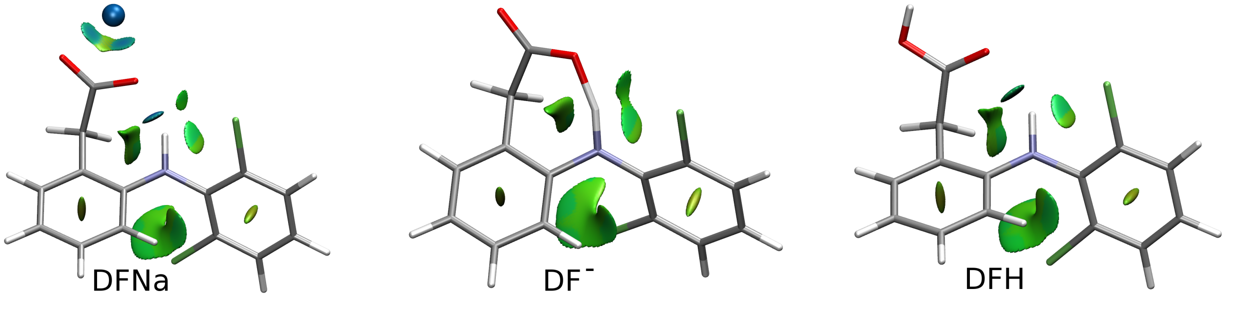


Figure S11: NCIPlot-calculated density »» for three investigated forms of diclofenac: DFNa, $DF^-$ and DFH

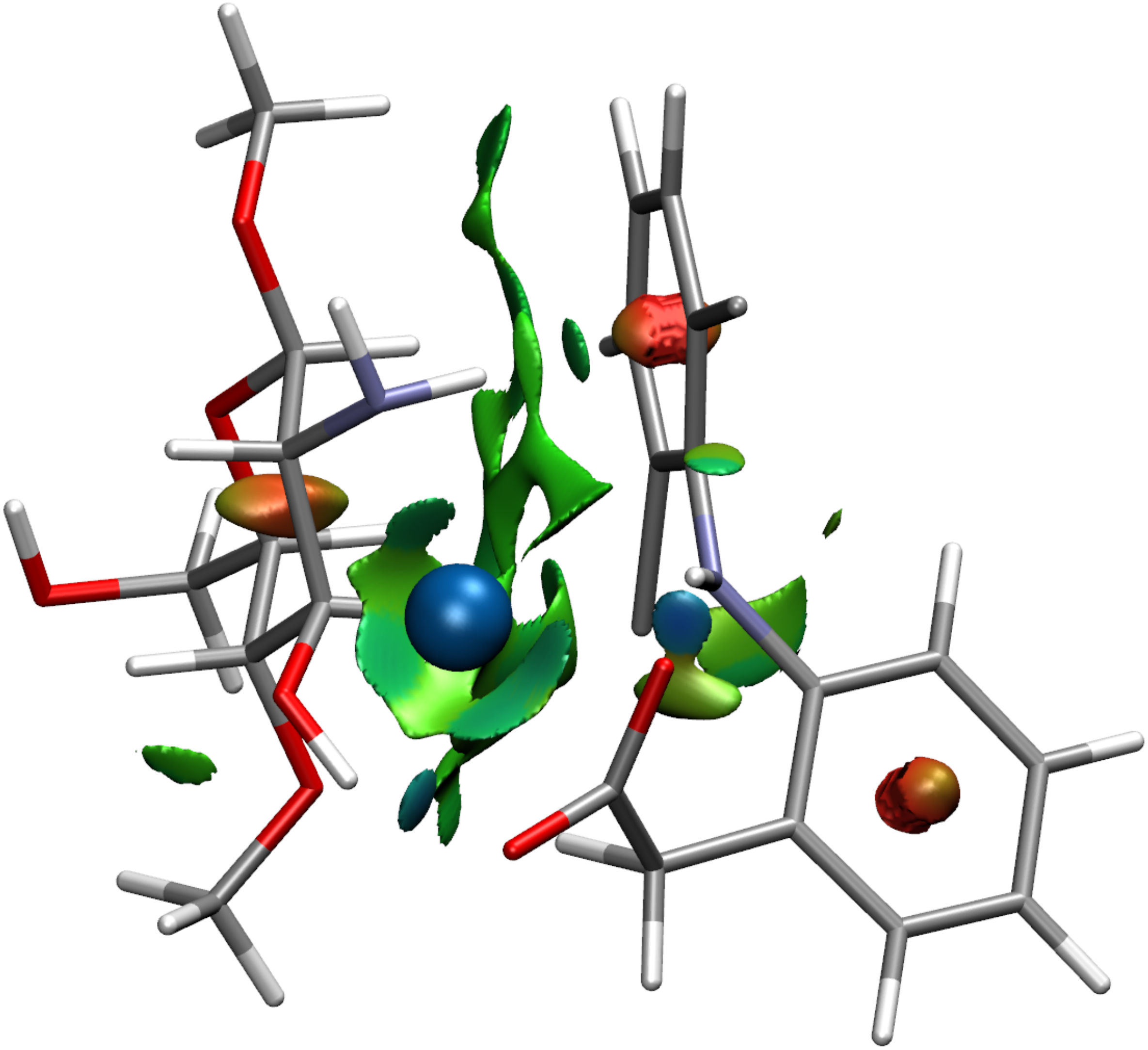

Figure S12: NCIPlot-calculated density »» for most stable structure **4**

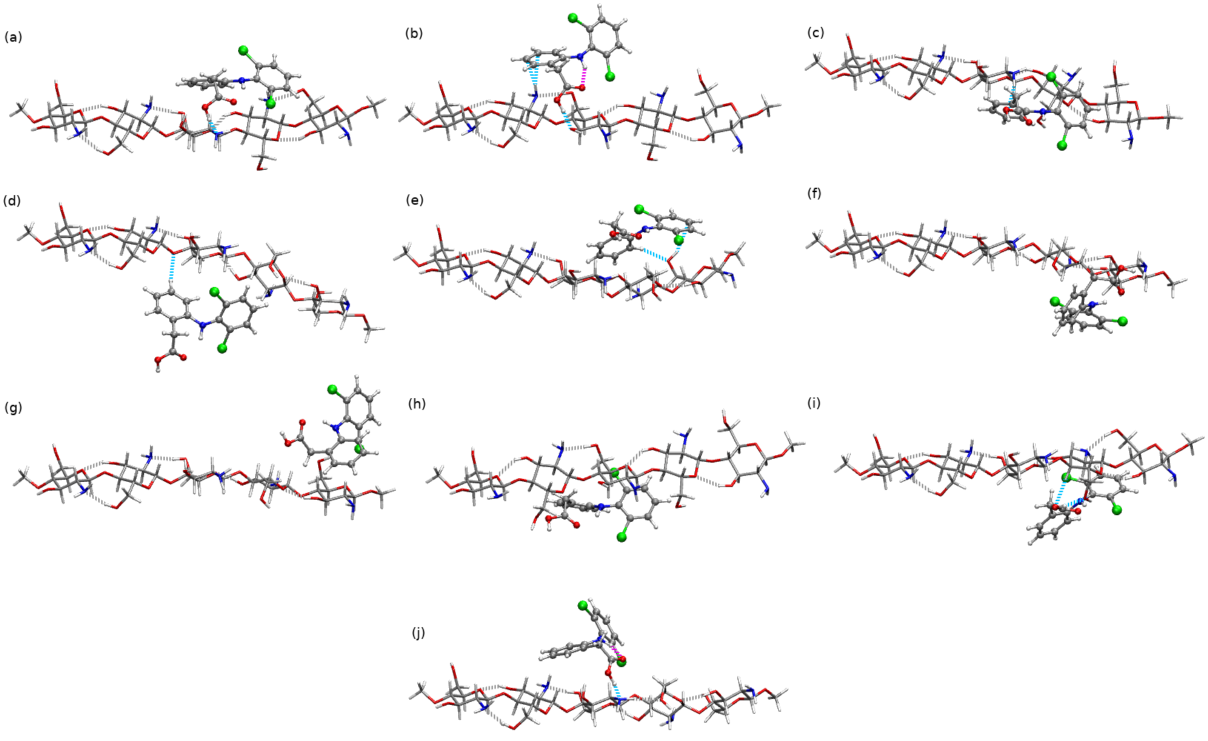


Figure S13: Optimized structures of CS5:DFH complexes (a) **1**, (b) **2**, (c) **3**, (d) **4**, (e) **5**, (f) **6**, (g) **7**, (h) **8**, (i) **9**, (j) **10**. Dashed lines depict hydrogen bonds (cyan: intermolecular, magenta: intramolecular in DFH, grey: intramolecular in CS5). CS5 presented in licorice representation and DFH with balls.

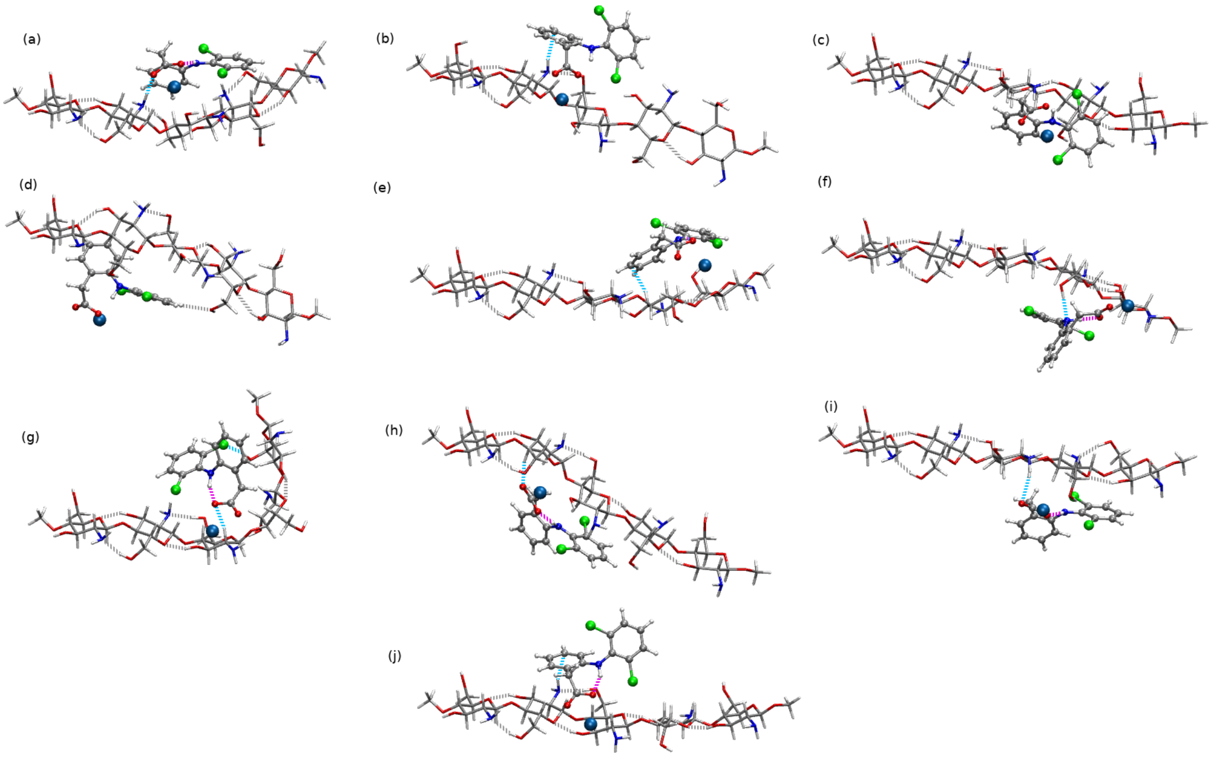


Figure S14: Optimized structures of CS5:DFNa complexes (a) **1**, (b) **2**, (c) **3**, (d) **4**, (e) **5**, (f) **6**, (g) **7**, (h) **8**, (i) **9**, (j) **10**. Dashed lines depict hydrogen bonds (cyan: intermolecular, magenta: intramolecular in DFNa, grey: intramolecular in CS5). CS5 presented in licorice representation and DFNa with balls.

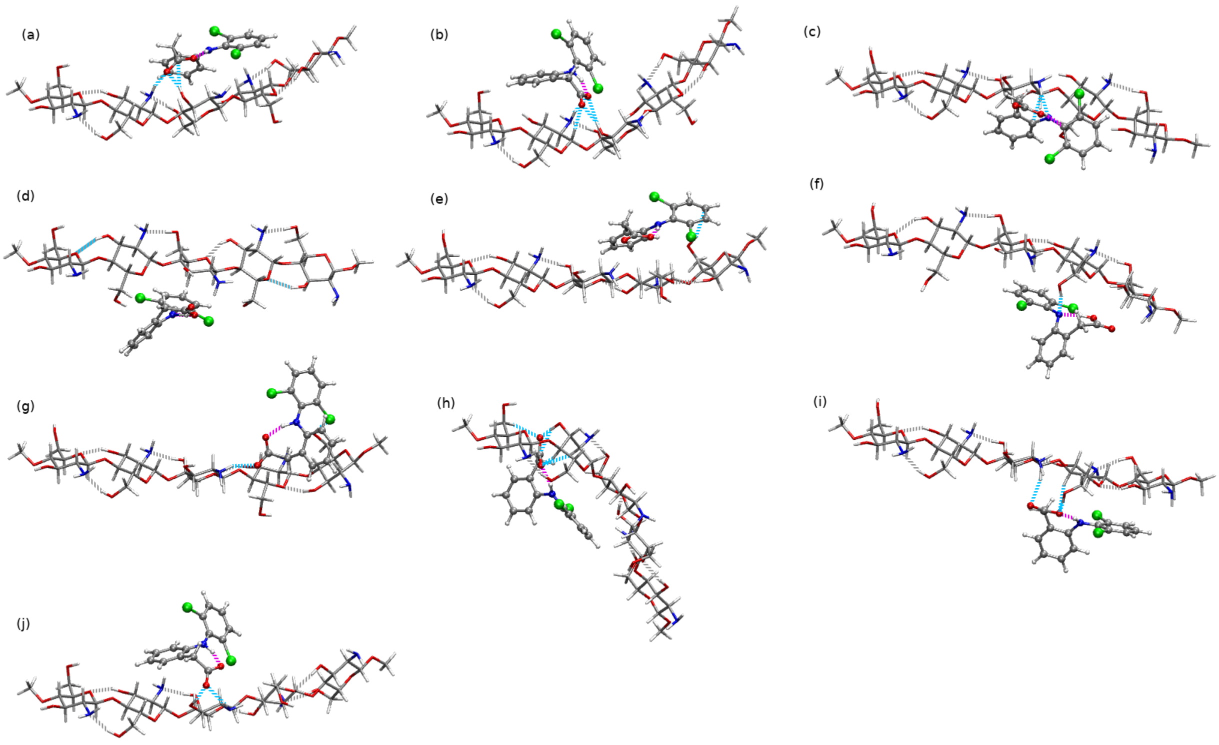


Figure S15: Optimized structures of CS5:DF$^-$ complexes (a) **1**, (b) **2**, (c) **3**, (d) **4**, (e) **5**, (f) **6**, (g) **7**, (h) **8**, (i) **9**, (j) **10**. Dashed lines depict hydrogen bonds (cyan: intermolecular, magenta: intramolecular in DF$^-$, grey: intramolecular in CS5). CS5 presented in licorice representation and DF$^-$ with balls.

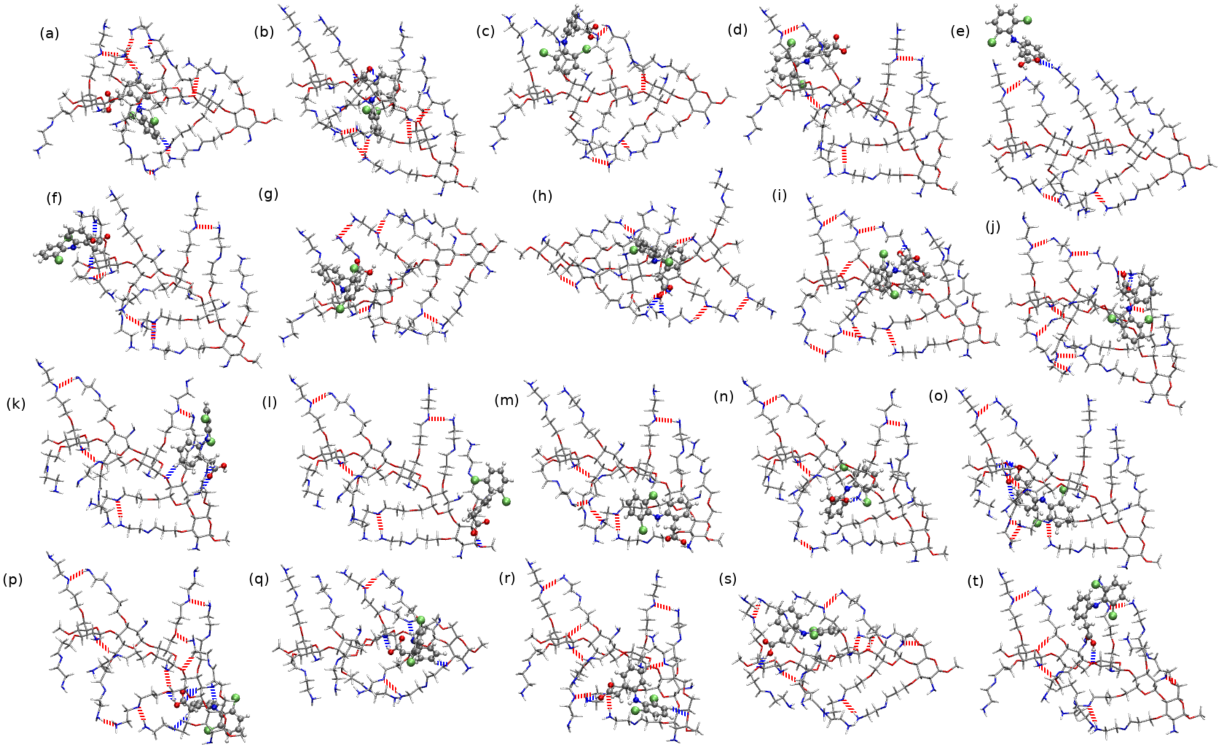


Figure S16: Optimized structures of $CS5(NH_2)_2$:DFH complexes (a) **1**, (b) **2**, (c) **3**, (d) **4**, (e) **5**, (f) **6**, (g) **7**, (h) **8**, (i) **9**, (j) **10**, (k) **11**, (l) **12**, (m) **13-PT**, (n) **14**, (o) **15**, (p) **16**, (q) **17**, (r) **18**, (s) **19**, (t) **20**. Dashed lines depict hydrogen bonds (blue: intermolecular, green: intramolecular in DFH, red: intramolecular in $CS5(NH_2)_2$). $CS5(NH_2)_2$ presented in licorice representation and DFH with balls.

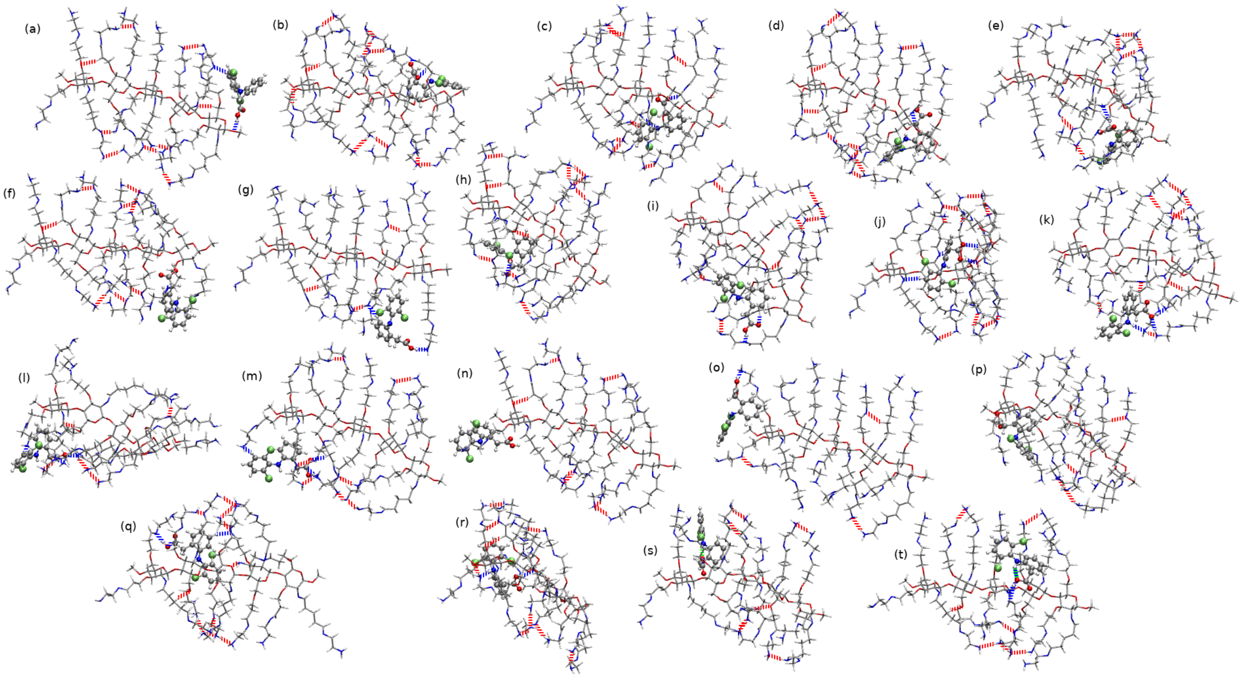


Figure S17: Optimized structures of $CS5(NH_2)_3$:DFH complexes (a) **1**, (b) **2**, (c) **3**, (d) **4**, (e) **5**, (f) **6**, (g) **7**, (h) **8**, (i) **9**, (j) **10**, (k) **11**, (l) **12**, (m) **13-PT**, (n) **14**, (o) **15**, (p) **16**, (q) **17**, (r) **18**, (s) **19**, (t) **20**. Dashed lines depict hydrogen bonds (blue: intermolecular, green: intramolecular in DFH, red: intramolecular in $CS5(NH_2)_3$). $CS5(NH_2)_3$ presented in licorice representation and DFH with balls.

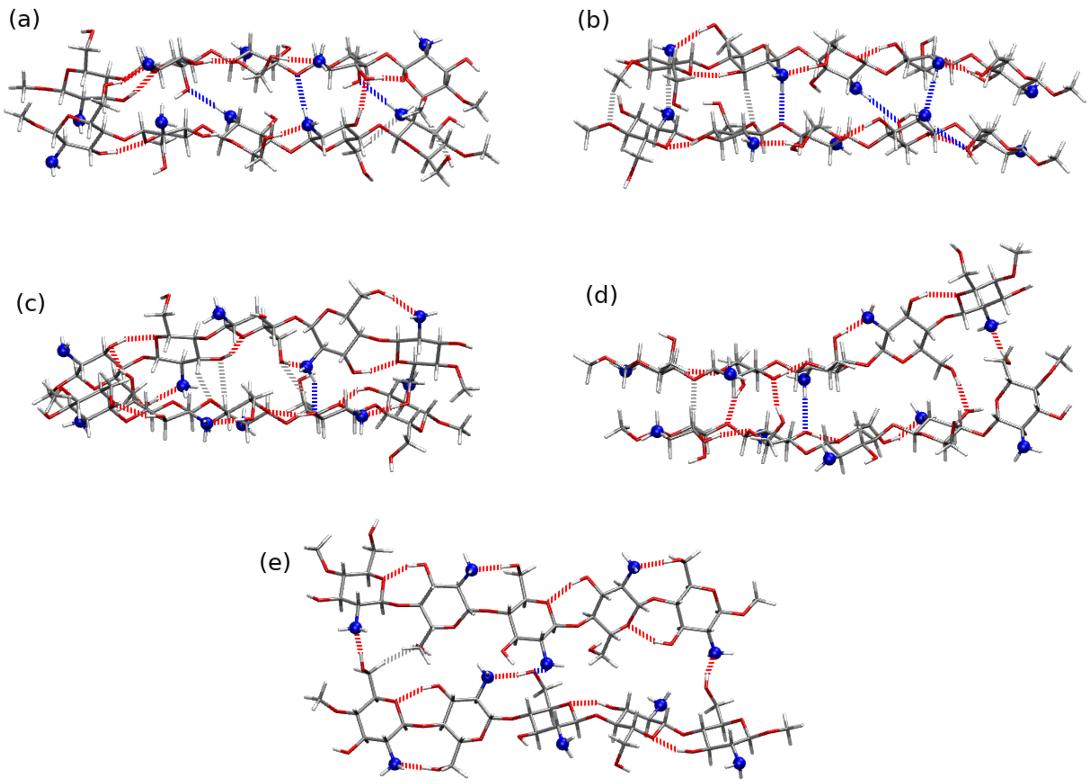


Figure S18: Optimized structures of the pristine $\left[CS5\right]_2$ dimers. Blue balls represent nitrogen atoms of the original amino groups close to the polymer backbone.

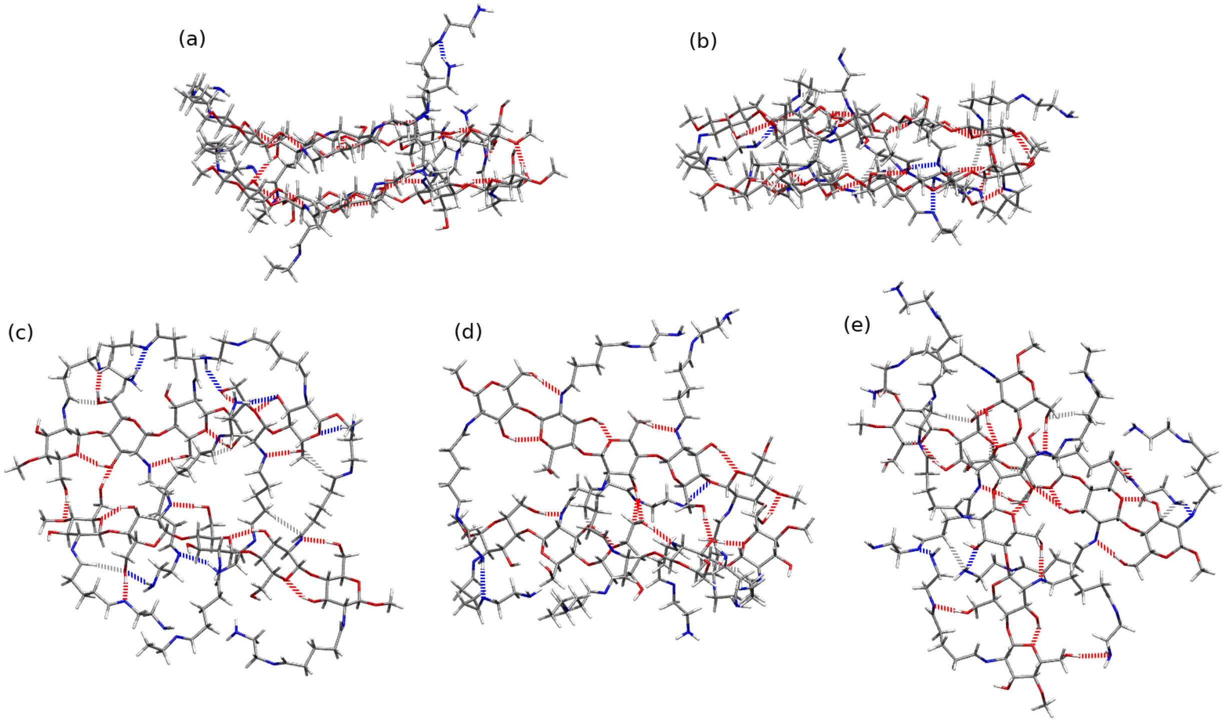


Figure S19: Optimized structures of $\left[CS5(NH_2)\right]$ dimers

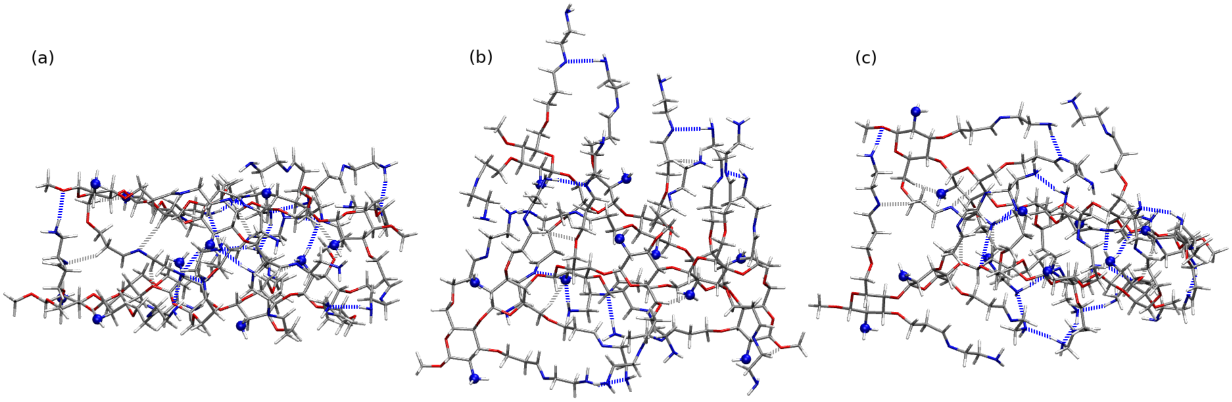


Figure S20: Optimized structures of $\left[CS5(NH_2)_2\right]_2$ dimers. Blue balls represent nitrogen atoms of the original amino groups close to the polymer backbone.

Table S2: Intramolecular N-H...O hydrogen bond parameters

| System | H...O | N...O | N-H | ∡N-H...O | ∡H-N...O |
|---|---|---|---|---|---|
| DFNa | | | | | |
| **1** | 1.68 | 2.66 | 1.05 | 155.0 | 15.5 |
| 2 | 3.07 | 3.21 | 1.02 | 88.7 | 72.8 |
| 3 | 1.90 | 2.83 | 1.03 | 147.5 | 21.2 |
| **4** | 1.78 | 2.72 | 1.03 | 149.0 | 19.7 |
| 5 | 1.74 | 2.71 | 1.04 | 153.6 | 16.6 |
| **6** | 1.87 | 2.81 | 1.03 | 148.5 | 20.4 |
| 7 | 1.81 | 2.73 | 1.03 | 146.9 | 21.2 |
| 7a | 1.93 | 2.92 | 1.03 | 161.2 | 12.2 |
| 8 | 1.80 | 2.72 | 1.04 | 146.4 | 21.4 |
| **9** | 1.71 | 2.70 | 1.04 | 156.4 | 14.7 |
| *10* | 1.84 | 2.73 | 1.03 | 141.5 | 24.9 |
| $DF^-$ | | | | | |
| 2 | 1.59 | 2.62 | 1.07 | 158.5 | 12.9 |
| **3** | 3.97 | 4.37 | 1.02 | 106.6 | 60.4 |
| **4** | 1.54 | 2.58 | 1.08 | 160.8 | 11.3 |
| 6 | 1.59 | 2.63 | 1.07 | 160.2 | 11.9 |
| **7a** | 4.41 | 4.05 | 1.02 | 62.9 | 104.2 |
| *8* | 1.52 | 2.57 | 1.08 | 161.6 | 10.7 |
| 9 | 1.59 | 2.63 | 1.07 | 160.2 | 11.9 |
| DFH | | | | | |
| 2 | 3.56 | 3.68 | 1.02 | 88.3 | 75.6 |
| *3* | 5.39 | 4.68 | 1.02 | 41.9 | 129.7 |
| **4** | 1.86 | 2.75 | 1.02 | 144.0 | 23.4 |
| 5 | 2.09 | 2.92 | 1.02 | 136.9 | 29.3 |
| **6** | 1.95 | 2.89 | 1.02 | 151.2 | 19.0 |
| 7 | 2.00 | 2.90 | 1.02 | 144.7 | 23.6 |
| 8 | 2.18 | 3.10 | 1.02 | 149.8 | 20.7 |
| **9** | 1.94 | 2.88 | 1.02 | 151.1 | 19.0 |

Table S3: Intramolecular N-H...Cl hydrogen bond parameters

| System | H...Cl | N...Cl | N-H | ∡N-H...Cl | ∡H-N...Cl |
|---|---|---|---|---|---|
| DFNa | | | | | |
| **1** | 2.64 | 3.03 | 1.05 | 149.7 | 58.5 |
| 2 | 2.49 | 3.01 | 1.02 | 110.5 | 51.0 |
| 3 | 2.74 | 3.03 | 1.03 | 96.2 | 64.0 |
| **4** | 2.63 | 3.02 | 1.03 | 102.6 | 57.9 |
| 5 | 2.64 | 3.04 | 1.04 | 103.0 | 57.6 |
| **6** | 2.57 | 3.03 | 1.03 | 106.3 | 54.5 |
| 7 | 3.08 | 2.97 | 1.03 | 74.1 | 86.4 |
| 7a | 2.70 | 3.05 | 1.03 | 99.7 | 60.9 |
| 8 | 3.07 | 2.99 | 1.03 | 75.7 | 84.7 |
| **9** | 2.68 | 3.02 | 1.04 | 98.5 | 61.5 |
| *10* | 2.97 | 2.97 | 1.03 | 80.0 | 80.0 |
| $DF^-$ | | | | | |
| 2 | 2.73 | 2.99 | 1.07 | 93.7 | 65.4 |
| **3** | 2.54 | 3.03 | 1.02 | 109.0 | 52.3 |
| **4** | 2.75 | 3.00 | 1.08 | 92.5 | 66.4 |
| 6 | 2.78 | 3.02 | 1.07 | 92.5 | 66.7 |
| **7a** | 2.39 | 3.01 | 1.02 | 118.5 | 44.2 |
| *8* | 2.74 | 3.00 | 1.08 | 92.8 | 66.1 |
| 9 | 2.78 | 3.02 | 1.07 | 92.5 | 66.7 |
| DFH | | | | | |
| 2 | 2.46 | 2.98 | 1.02 | 111.4 | 50.0 |
| *3* | 2.46 | 3.03 | 1.02 | 114.8 | 47.4 |
| **4** | 2.60 | 3.01 | 1.02 | 103.4 | 57.3 |
| 5 | 2.52 | 3.01 | 1.02 | 108.9 | 52.4 |
| **6** | 2.68 | 3.01 | 1.02 | 99.0 | 61.4 |
| 7 | 2.47 | 2.99 | 1.02 | 110.5 | 50.8 |
| 8 | 2.71 | 3.03 | 1.02 | 98.1 | 62.4 |
| **9** | 2.64 | 3.02 | 1.02 | 101.7 | 58.9 |

Table S4: SAPT0 interaction energy components for the CS5:DFH (boldfaced most attractive SAPT0 interaction; boxes for ELST/DISP ratio smaller than 0.59, denoting dispersion-dominated systems and underlined energies for ELST/DISP ratio exceeding 1.7, denoting electrostatic-dominated systems)

| System | ELST | EXCH | IND | DISP | SCS-DISP | HF | SAPT0 | SCS-SAPT0 | ELST/DISP | ELST/SCS-DISP |
|---|---|---|---|---|---|---|---|---|---|---|
| cc-pVDZ | | | | | | | | | | |
| **1** | -46.51 | 67.06 | -18.82 | -32.73 | -25.41 | 1.73 | **-31.00** | -23.68 | 1.42 | 1.83 |
| 2 | -31.31 | 44.20 | -13.73 | -20.38 | -15.80 | -0.85 | -21.23 | -16.64 | 1.54 | 1.98 |
| 3 | -13.17 | 22.73 | -3.63 | -17.08 | -13.21 | 5.93 | -11.15 | -7.28 | 0.77 | 1.00 |
| 4 | -12.30 | 20.59 | -3.84 | -16.21 | -12.52 | 4.44 | -11.77 | -8.07 | 0.76 | 0.98 |
| 5 | -20.03 | 34.73 | -5.73 | -28.08 | -21.69 | 8.97 | -19.11 | -12.72 | 0.71 | 0.92 |
| 6 | -11.49 | 23.11 | -3.04 | -20.44 | -15.78 | 8.58 | -11.86 | -7.20 | 0.56 | 0.73 |
| 7 | -9.06 | 18.34 | -2.78 | -14.81 | -11.43 | 6.49 | -8.32 | -4.94 | 0.61 | 0.79 |
| 8 | -8.93 | 20.10 | -2.15 | -13.33 | -10.28 | 9.02 | -4.31 | -1.26 | 0.67 | 0.87 |
| 9 | -26.50 | 39.45 | -8.55 | -21.83 | -16.90 | 4.40 | -17.43 | -12.50 | 1.21 | 1.57 |
| **10** | -42.14 | 58.91 | -24.07 | -21.75 | -16.93 | -7.31 | **-29.06** | -24.24 | 1.94 | 2.49 |
| aug-cc-pVDZ | | | | | | | | | | |
| **1** | -45.25 | 66.73 | -19.84 | -43.04 | -33.68 | 1.65 | **-41.39** | -32.03 | 1.05 | 1.34 |
| 2 | -30.83 | 44.02 | -14.37 | -28.04 | -21.90 | -1.18 | -29.22 | -23.08 | 1.10 | 1.41 |
| 3 | -12.66 | 22.53 | -3.91 | -22.83 | -17.81 | 5.96 | -16.88 | -11.85 | 0.55 | 0.71 |
| 4 | -11.91 | 20.50 | -3.92 | -21.61 | -16.83 | 4.67 | -16.94 | -12.16 | 0.55 | 0.71 |
| 5 | -19.44 | 34.53 | -5.93 | -38.39 | -29.91 | 9.16 | -29.24 | -20.76 | 0.51 | 0.65 |
| 6 | -11.11 | 22.86 | -3.18 | -28.44 | -22.13 | 8.56 | -19.88 | -13.57 | 0.39 | 0.50 |
| 7 | -8.72 | 18.29 | -3.01 | -20.45 | -15.92 | 6.55 | -13.90 | -9.36 | 0.43 | 0.55 |
| 8 | -8.69 | 20.05 | -2.27 | -18.27 | -14.21 | 9.08 | -9.19 | -5.13 | 0.48 | 0.61 |
| 9 | -25.67 | 39.18 | -9.01 | -29.80 | -23.25 | 4.51 | -25.29 | -18.75 | 0.86 | 1.10 |
| **10** | -41.22 | 58.69 | -25.03 | -28.92 | -22.66 | -7.57 | **-36.49** | -30.23 | 1.43 | 1.82 |

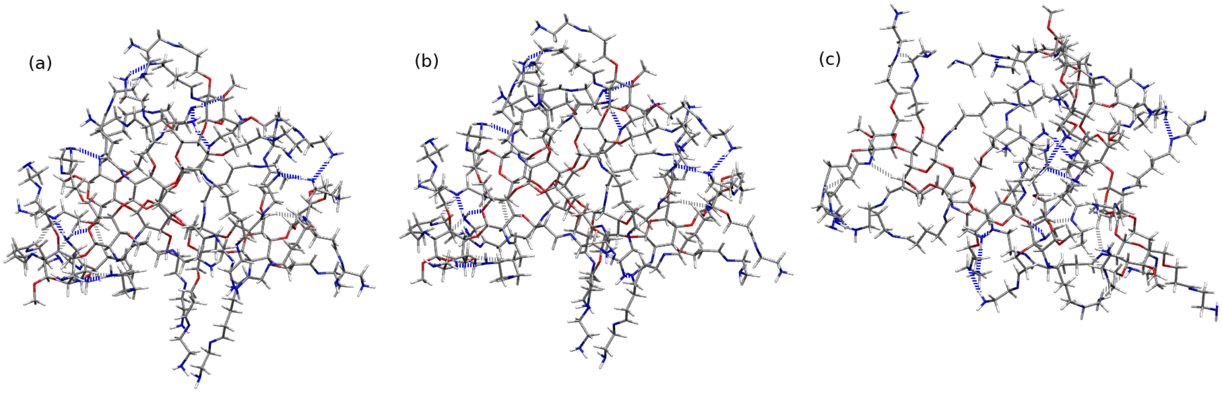


Figure S21: Optimized structures of $\left[CS5(NH_2)_3\right]_2$ dimers
